# TYK2 regulates tau levels, phosphorylation and aggregation in a tauopathy mouse model

**DOI:** 10.1038/s41593-024-01777-2

**Published:** 2024-11-11

**Authors:** Jiyoen Kim, Bakhos Tadros, Yan Hong Liang, Youngdoo Kim, Cristian Lasagna-Reeves, Jun Young Sonn, Dah-eun Chloe Chung, Bradley Hyman, David M. Holtzman, Huda Yahya Zoghbi

**Affiliations:** 1 Department of Molecular and Human Genetics, Baylor College of Medicine, Houston, TX USA; 2 Jan and Dan Duncan Neurological Research Institute at Texas Children’s Hospital, Houston, TX USA; 3 Stark Neurosciences Research Institute and Department of Anatomy, Cell Biology & Physiology, Indiana University School of Medicine, Indianapolis, IN USA; 4 Neurology at Harvard Medical School and Massachusetts General Hospital, Boston, MA USA; 5 Department of Neurology, Hope Center for Neurological Disorders, Knight Alzheimers’ Disease Research Center, Washington University in St. Louis, St. Louis, MO USA; 6 Departments of Neuroscience, Pediatrics, and Neurology, Baylor College of Medicine, Houston, TX USA; 7 Howard Hughes Medical Institute, Chevy Chase, MD USA

**Keywords:** Molecular neuroscience, Phosphorylation, Alzheimer's disease

## Abstract

Alzheimer’s disease is one of at least 26 diseases characterized by tau-positive accumulation in neurons, glia or both. However, it is still unclear what modifications cause soluble tau to transform into insoluble aggregates. We previously performed genetic screens that identified tyrosine kinase 2 (TYK2) as a candidate regulator of tau levels. Here we verified this finding and found that TYK2 phosphorylates tau at tyrosine 29 (Tyr29) leading to its stabilization and promoting its aggregation in human cells. We discovered that TYK2-mediated Tyr29 phosphorylation interferes with autophagic clearance of tau. We also show that TYK2-mediated phosphorylation of Tyr29 facilitates pathological tau accumulation in P301S tau-transgenic mice. Furthermore, knockdown of *Tyk2* reduced total tau and pathogenic tau levels and rescued gliosis in a tauopathy mouse model. Collectively, these data suggest that partial inhibition of TYK2 could thus be a strategy to reduce tau levels and toxicity.

## Abstract (web summary)

Tyrosine kinase 2 (TYK2) critically regulates tau levels and aggregation by phosphorylating tau’s tyrosine 29. Partial inhibition of TYK2 mitigates tau pathologies in cells and mice, highlighting TYK2 as a potential therapeutic target for Alzheimer’s disease and other tauopathies.

## Main

Over two dozen different diseases have been identified so far whose hallmark neuropathological feature is the presence of neuronal and/or glial accumulations of tau protein^1^. Tau is a predominantly neuronal protein that binds to tubulin to promote assembly of the microtubule network that underpins intracellular transport. Its function is regulated by numerous post-translational modifications, such as phosphorylation, ubiquitination, methylation and acetylation^2^. In pathological states, tau protein undergoes aberrant modifications—predominantly hyperphosphorylation—then dissociates from microtubules, misfolds, propagates to neighboring cells and accumulates into intracellular neurofibrillary tangles (NFTs)^2–5^. Disturbed tau function would be expected, then, to have wide-ranging effects in the brain, and tauopathies do usually affect cognition, language, behavior and movement to varying degrees, as is the case with Alzheimer’s disease (AD), frontotemporal dementia due to tauopathy, progressive supranuclear palsy and corticobasal degeneration^6,7^.

Because human and mouse studies point to tau concentrations as one of the determining factors of disease initiation and progression^8–10^, we performed cross-species genetic screens to identify candidate regulators of tau levels^11,12^. One of these screens yielded tyrosine kinase 2 (TYK2), a member of the Janus kinases protein family that phosphorylates tyrosine residues on substrate proteins^13^. TYK2 is known for its association with the cytoplasmic domain of type I and II cytokine receptors to propagate cytokine signals by phosphorylating receptor subunits^13–15^. TYK2 may, indeed, have a role in neuroinflammation in neurodegenerative diseases such as AD^16^. Furthermore, we were intrigued by our finding that knocking down TYK2 reduced endogenous tau levels in human cells as tau phosphorylation by tyrosine kinases is important in the pathogenesis of tauopathies, including AD^17–19^—an increase in tau phosphorylation on tyrosine residues correlates with the formation and accumulation of toxic tau species^20,21^. All five of tau’s tyrosine residues (Y18, Y29, Y197, Y310 and Y394; numbered according to the longest central nervous system isoform of tau) are subject to phosphorylation. Fyn and LcK (lymphocyte-specific protein tyrosine) kinase, a member of the Src kinase family, can phosphorylate tau at all five tyrosines in vitro or in cell culture, but they are more prone to phosphorylate tau on Tyr18 (refs. ^22,23^). Tyr18 residue is also reported to be phosphorylated by spleen tyrosine kinase^24^ and proline-rich tyrosine kinase 2 (Pyk2; ref. ^21^). Tau tubulin kinase 1 phosphorylates tau at Tyr197 in vitro^25,26^, and members of the c-ABL kinase family (c-Abl and Arg kinase) phosphorylate Tyr394 (refs. ^27,28^). Notably, details in ref. ^19^ showed that depleting Fyn reduced tau Tyr18 phosphorylation and led to near-complete ablation of NFTs in tau-transgenic mice. These findings suggest that tyrosine phosphorylation has a critical role in regulating tau accumulation and might influence the course of tau-driven neurodegeneration. So far, no dedicated kinases for phosphorylating Y29 or Y310 have been identified.

In the present study, we find that TYK2 phosphorylates tau protein at tyrosine 29 (Tyr29) residue (Y29) and stabilizes its levels in human cells and cultured mouse primary neurons. TYK2 enhances the aggregation of pathogenic tau (tau441-P301S), except when Y29 is mutated to phenylalanine, preventing phosphorylation (tau441-Y29F-P301S). We also find that TYK2 phosphorylation at Y29 facilitates nitration at the same residue. This sheds light on earlier reports that in human tauopathies, nitrated Y29 stabilizes filaments and destabilizes microtubules^29–31^. Notably, we show that knockdown of *Tyk2* in the P301S transgenic mouse model of tauopathy reduces pathogenic, hyperphosphorylated tau as well as total amounts of tau protein.

## Results

### TYK2 regulates tau concentrations in cells and mouse brains

**Extended Data Fig. 1:**
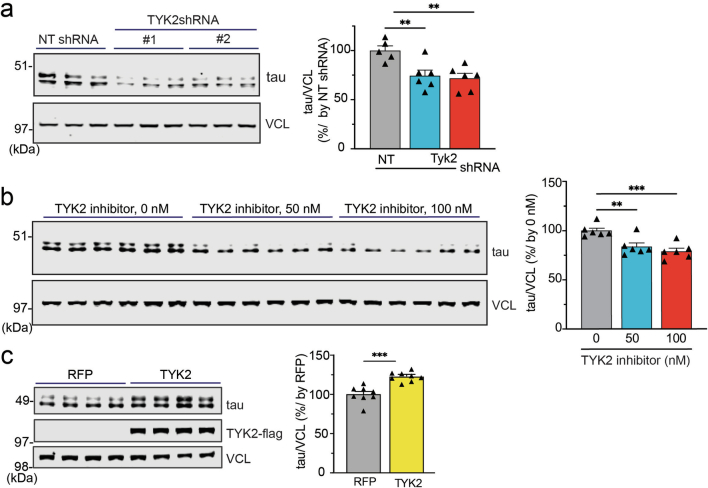
TYK2 regulates tau concentrations in cells. **a**–**c**, Immunoblotting (IB) image and quantitative graph showing reduction of tau levels upon either TYK2 knockdown using shRNA (n = 6, one-way ANOVA, Dunnett’s multiple comparison test, P_(NT-TYK2 shRNA1)_ = 0.0087, P_(NT-TYK2 shRNA2)_ = 0.0045; **a**) or TYK2 catalytic inhibition using a small molecule (deucravacitinib, BMS986165; n = 6, one-way ANOVA, Dunnett’s multiple comparison test, P_(DMSO-TYK2 inhibitor, 50 nM)_ = 0.0058, P_(DMSO-TYK2 inhibitor, 100 nM)_ = 0.0007; **b**). In contrast, TYK2 overexpression increases endogenous tau levels in a neuroblastoma cell line (n = 8, unpaired t test, two-tailed, P = 0.0001; **c**). n is the number of biological replicates. Data are represented as mean ± SEM (**p < 0.01, ***p < 0.0005). Source data

First, we validated if TYK2, a candidate from our previous screen^11^, is a true regulator of tau. In human neuroblastoma cell lines (BE(2)-C and SH-SY5Y), we could reduce the endogenous levels of tau by reducing TYK2 function, either by infection with lentivirus containing a short hairpin RNA (shRNA) that suppresses TYK2 expression or by enzymatic inhibition of TYK2 using a small molecule (deucravacitinib; Extended Data Fig. 1a,b). Conversely, TYK2 overexpression by infection with lentivirus containing *TYK2* increased endogenous tau levels (Extended Data Fig. 1c). TYK2 activity thus contributes to the regulation of endogenous tau levels in human cells.

**Fig. 1:**
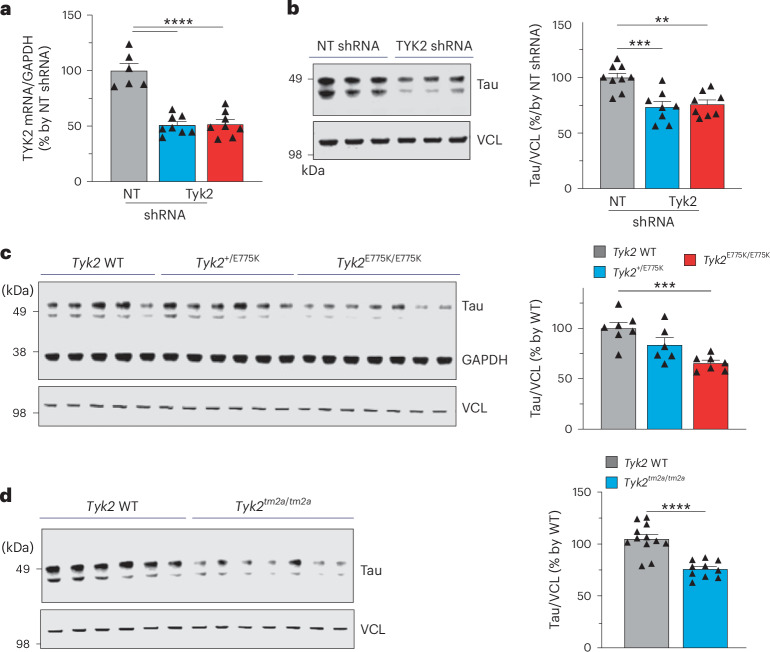
Genetically decreasing Tyk2 in mice reduces tau levels. **a**, The level of *Tyk2* mRNA in mouse brain determined by qPCR at 3 weeks after ICV injection of AAV8 harboring *Tyk2* shRNA (*n* = 8) or NT shRNA (*n* = 6; one-way ANOVA, Bonferroni post hoc tests, *P* < 0.0001). **b**, IB image and quantification of tau levels showing that TYK2 knockdown by shRNA reduced endogenous tau levels in the mouse brain at 3 weeks of age (*n* = 8–9; one-way ANOVA, Bonferroni post hoc tests, *P*_(NT versus *Tyk2*shRNA1)_ = 0.0004, *P*_(NT versus *Tyk2*shRNA2)_ = 0.0011). **c**, IB image and quantification of endogenous tau protein in the brain from mice containing heterozygous or homozygous mutant *Tyk2* E775K, or WT littermates at 1 month old (*n* = 7; one-way ANOVA, Bonferroni post hoc tests, *P*_(WT versus *Tyk2*_E2775K/+)_ = 0.0853, *P*_(WT versus *Tyk2*_E2775K/E2775K)_ = 0.0004). **d**, IB image and quantification of endogenous tau protein in the brain from homozygous Cre-inducible *Tyk2* KO mice (*Tyk2*^*tm2a*^^/^^*tm2a*^) at 1 month after P0-ICV injection of AAV8-Cre (*n* = 10–12; unpaired *t* test/two-tailed, *P* = 0.000018). ‘*n*’ is the number of mice. Data are represented as mean ± s.e.m., **P < 0.01, ***P < 0.0005 and ****P < 0.0001. VCL, vinculin. Source data

Next, we examined the effect of Tyk2 on tau levels in the mouse brain. For this, we delivered adeno-associated virus serotype 8 (AAV8) harboring *Tyk2* shRNA into the cerebral ventricles of neonatal mice (postnatal day 0 or P0) via intracerebroventricular (ICV) injection^12^. Bilateral injection of recombinant AAV8 at 6 × 10^10^ particles per hemisphere suppressed *Tyk2* to ≤40–50% of wild-type (WT) levels in the mouse brain (Fig. 1a) without discernible toxicity at the time of tissue collection. Immunoblotting (IB) analysis of brain homogenates from the injected mice revealed that *Tyk2* knockdown reduced endogenous tau levels by around 25% (Fig. 1b).

We found similar results using the *Tyk2*^E775K^ mouse, in which Tyk2 protein is destabilized and no protein is detected despite the presence of mRNA transcripts^32^; endogenous tau was reduced more in the homozygous *Tyk2*^E775K/E775K^ than the heterozygous mutant mice (Fig. 1c). In addition, P0-ICV injection of AAV8 harboring Cre into Cre-dependent *Tyk2* mice (*Tyk2*^*tm2a*/*tm2a*^) led to a significant reduction in tau protein levels in the adult brains (Fig. 1d). We achieved similar reductions of tau in the two homozygous mouse models—*Tyk2*^E775K/E775K^ (*Tyk2* knockdown in all cell types) and *Tyk2*^*tm2a*/*tm2a*^ (*Tyk2* knockdown mostly in neurons and a subset of astrocytes)—suggesting that *Tyk2* knockdown predominantly affects neurons rather than non-neuronal cells.

### TYK2 phosphorylates and stabilizes tau in a phosphorylation-dependent manner

**Extended Data Fig. 2:**
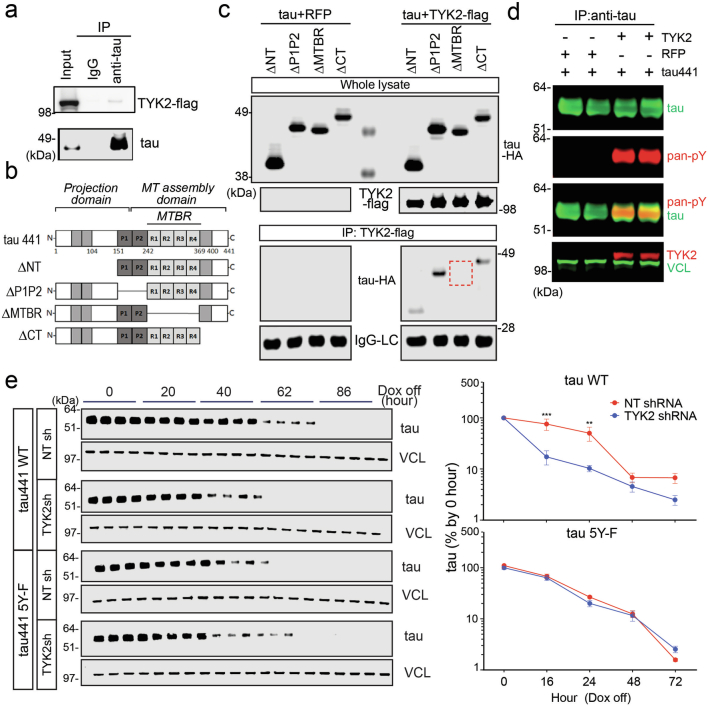
TYK2 binds to and phosphorylates tau protein. **a**, Representative co-immunoprecipitation (IP) image (3 repeats) showing an interaction between tau and TYK2-flag in HEK293T cells. **b**, Schematic diagram of the truncated tau proteins. **c**, Representative Co-IP of the individual truncated tau proteins after IP of TYK2-flag in HEK293T cells (2 repeats). All tau fragments except truncated MTBR (red dotted box) bind to TYK2. **d**, Representative tau-IP image (4 repeats) showing phosphorylation of tau at Tyr residue(s) in the presence of TYK2, detected by total tau antibody and pan-phospho-Tyr antibody. **e**, IB image and quantification graphs showing degradation of WT tau or tau with all its Tyr residues mutated after knockdown of *Tyk2* in mouse primary cultured neurons (n = 4, n is the number of individual treatments). Lentivirus containing genes encoding given proteins under a TTA-inducible promoter was used to transduce cells. Tau protein expression was induced by DOX treatment for 24 hours, after which DOX was removed and cells were collected at indicated time points. The cell lysates were subjected to IB analysis. NT, N-terminal region of tau (amino acids 1–151), P1P2, proline-rich region (amino acids 152–242), CT, C-terminal region of tau (amino acid 370–441), MTBR, microtubule-binding repeats (amino acids 243–400). n is the number of biological replicates. Data are represented as mean ± SEM (**p < 0.01, ***p < 0.0005). Source data

To verify that TYK2 binds to and phosphorylates tau protein, we performed immunoprecipitation (IP) of endogenous tau protein in HEK293T cells. The tau-IP pulled down transfected TYK2, indicating that tau and TYK2 interact in cells (Extended Data Fig. 2a). To determine which tau protein domains mediate the interaction with TYK2, we conducted co-IP assays in HEK293T cells expressing TYK2-flag and individually expressing different tau fragments (Extended Data Fig. 2b). IB analysis of tau-IP samples showed that tau’s microtubule-binding repeat region is required for TYK2 binding (Extended Data Fig. 2c). Next, we conducted an in vitro cellular kinase assay, performing IP on cells expressing TYK2 and tau441(2N4R tau) and immunoblotting the IP samples using pan-phospho-tyrosine antibodies. IB analysis of tau-IP samples revealed that tau protein was phosphorylated on tyrosine residues in the presence of TYK2 (Extended Data Fig. 2d).

To test whether TYK2 stabilizes tau through phosphorylation, we replaced all five tyrosines (Y18, Y29, Y197, Y310 and Y394) with phenylalanine, generating a mutant form of tau (tau441_5Y-F) that cannot be phosphorylated on any tyrosine, and expressed it under the tetracycline transactivator (TTA)-dependent promoter. We analyzed the protein turnover rate of tau441 WT and tau441_5Y-F and found that *TYK2* knockdown by a lentivirus harboring *TYK2* shRNA promoted the degradation of tau441 WT but not tau441_5Y-F (Extended Data Fig. 2e). These data suggested that TYK2 phosphorylation on tyrosines stabilizes tau protein in human cells. The next step was to determine which tyrosine residues were involved.

### TYK2 stabilizes tau protein through Tyr29 phosphorylation

**Fig. 2:**
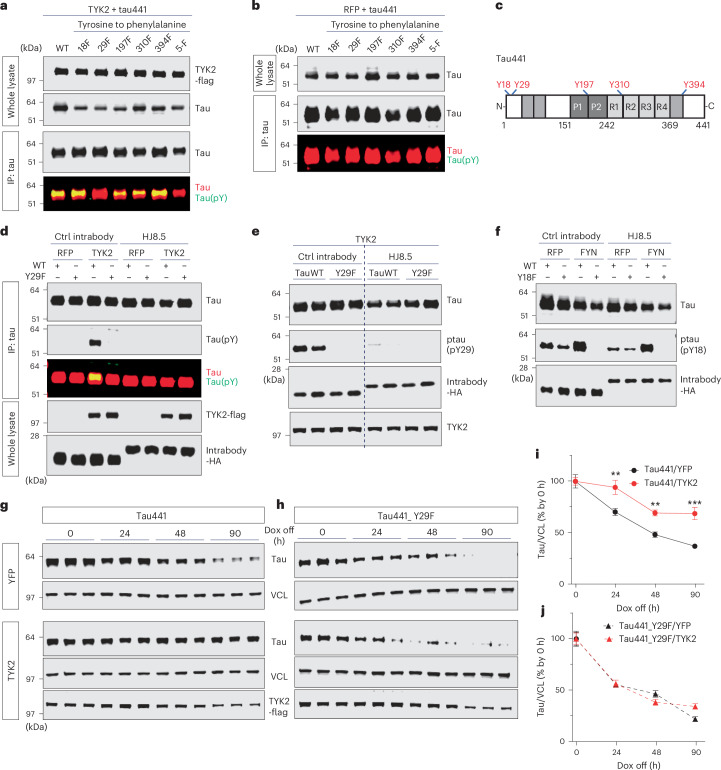
TYK2 phosphorylates tau at Tyr29 residue and thus stabilizes tau protein. **a**,**b**, IB image of the phosphorylation of tau at Tyr residues in the presence (**a**) or absence (**b**) of TYK2 in HEK293T cells. Tau protein was collected by IP and detected by pan-phospho-Tyr antibody. p-Tyr was observed only in the presence of TYK2 and was lost in the phosphorylation disabling mutation at Tyr29 (tau441-Y29F) indicated by loss of p-Tyr signal despite the addition of TYK2. **c**, Schematic diagram of Tyr residues of tau441 protein. **d**, IB image of p-Tyr signal detected in neither tau441-Y29F nor tau treated with tau intrabody masking Tyr29 on tau protein in HEK293T cells. Either tau441 or tau441-Y29F was co-expressed with TYK2 in HEK293T cells that stably expressed either tau intrabody (clone HJ8.5, targeting tau amino acid residues 25–30) or control intrabody (clone HJ15.4). **e**,**f**, IB image of ptau detected by our ptau (pY29) antibody (**e**) or ptau (pY18) antibody (**f**) in the indicated samples in the presence or absence of tau intrabody. **g**–**j**, IB images and quantification of tau showing the turnover rate of tau441 (**g** and **i**) and tau441-Y29F (**h** and **j**; *n* = 6, *n* is the number of biological repeats). Mouse primary cultured cortical neurons were virally infected with genes encoding tau441 WT or tau441-Y29F under a TTA-inducible promoter. Tau protein was expressed by DOX treatment for 24 h, after which DOX was removed and cells were collected at indicated time points. The blots are representative of three independent experiments. **P < 0.005, ***P < 0.0005 versus vector-expressing control. Data presented as mean ± s.e.m., two-way ANOVA/Dunnett’s test. 5-F, tau with all five Tyr converted to Phe. Source data

**Extended Data Fig. 3:**
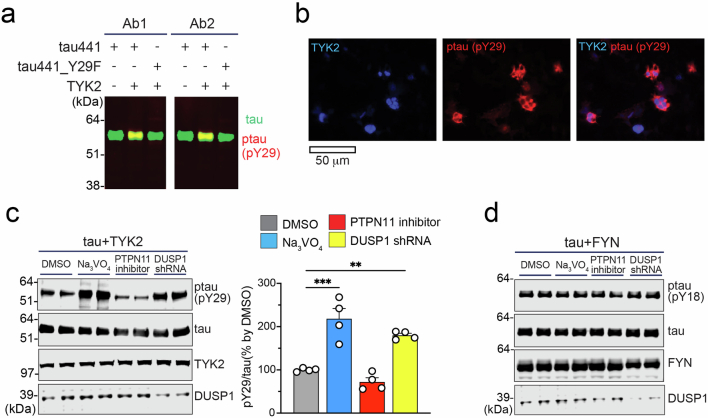
Validation of our newly generated tau-pY29 antibody for identification of a phosphatase that dephosphorylates tau at Tyr29. **a**, IB image showing that our newly created phospho-tau (pY29) antibody (two independent bleedings) selectively detects TYK2-dependent phosphorylation on tau, but not on RFP control protein or tau441_Y29F. **b**, Representative immunocytochemistry image of HEK293T cells with TYK2 overexpression, showing TYK2 (detected by flag antibody, left) co-localizes with pTyr29 by the new phospho-tau (pY29) antibody (left and right; 12 individual images from 3 independent experiments). **c**, IB image of TYK2-mediated phospho-tau (pY29, left) and its quantification in 293T cells treated as indicated (n = 4, n is the number of biological replicates, one-way ANOVA, Sidak’s multiple comparison test, P_(DMSO-Na4VO4)_ = 0.000103, P_(DMSO-PTPN11 inhibitor)_ = 0.40018, P_(DMSO-DUSP1shRNA)_ = 0.002961). **d**, Representative IB image of FYN-mediated Tyr18 in HEK293T cell treated as indicated (2 independent experiments). Na_3_VO_4_, general inhibitor of tyrosine kinase; batoprotafib, PTPN11 inhibitor (TNO155, 100 nM, for 48 h). Data are represented as mean ± SEM (**p < 0.01, ***p < 0.0005). Source data

To determine which tyrosine residues are involved in TYK2-mediated phosphorylation, we substituted each tyrosine residue with phenylalanine and performed an in vitro kinase assay in HEK293T cells. TYK2 phosphorylated all tau variants except for tau containing a Y29F mutation (Fig. 2a–c). To confirm that TYK2 phosphorylates tau at Tyr29, we repeated the in vitro kinase assay in stable cell lines that express either an antitau intrabody, HJ8.5, or a control intrabody. HJ8.5 is one of the antitau intrabodies that target residues 25–30 of tau^33^, spanning Tyr29. Masking this Tyr29 residue with the HJ8.5 intrabody almost completely blocked tau phosphorylation by TYK2 (Fig. 2d). Given this result, we generated a phospho-tau (p-Tyr29) antibody that detected tau specifically phosphorylated at Tyr29 residue. This antibody detected tau in the presence of TYK2 but not tau441-Y29F in IB assays (Extended Data Fig. 3a). Our newly generated antibody also detected phospho-tau by TYK2 in HEK293T cells (Extended Data Fig. 3b). We validated phospho-tau (p-Tyr29) antibody using intrabody HJ8.5, showing that p-Tyr29 detects TYK2-phosphorylated tau in the presence of the control intrabody but not tau441-Y29F or when Tyr29 is masked by HJ8.5 (Fig. 2e). In contrast, phospho-tau (pTyr18) antibodies detected the FYN-phosphorylated tau regardless of the intrabody (Fig. 2f).

Recognizing that the signal of phosphorylated tau at Tyr29 was enhanced considerably by treating the cells with a general inhibitor of tyrosine phosphatase (Na_3_VO_4_), we sought to discover which phosphatase dephosphorylates p-Tyr29. We inhibited either protein tyrosine phosphatase nonreceptor type 11 (PTPN11) or dual specificity protein phosphatase 1 (DUSP1), both of which interact with tau protein^34,35^. Inhibition of DUSP1 but not PTPN11 led to increasing TYK2-mediated Tyr29 phosphorylation (Extended Data Fig. 3c). Neither the inhibition of PTPN11 nor the inhibition of DUSP1 affected FYN-mediated Tyr18 phosphorylation (Extended Data Fig. 3d). These data suggest that DUSP1 dephosphorylates p-Tyr29.

To examine whether TYK2 stabilizes tau by phosphorylating the Tyr29 residue, we virally transduced primary cultured mouse cortical neurons with either the tetracycline-inducible tau441 WT or the mutant form of tau (tau441-Y29F). We then treated the cells with doxycycline (DOX; 100 nM) for 24 h to induce tau expression. After blocking de novo protein synthesis by removing DOX, we collected cells at several different time points up to 72 h. Here we found that TYK2 expression stabilized the tau441 protein (Fig. 2g,i) but had no effect on tau441-Y29F (Fig. 2h,j). These data strongly suggest that TYK2-mediated Tyr29 phosphorylation stabilizes tau.

**Fig. 3:**
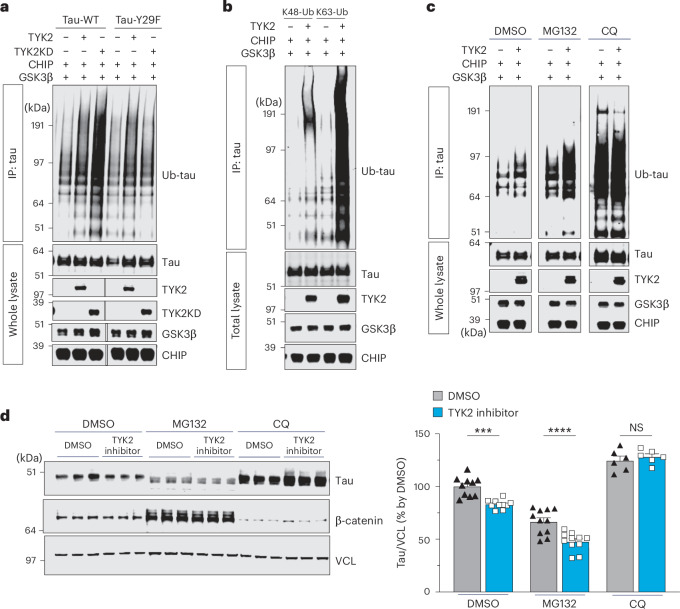
TYK2-mediated Tyr29 phosphorylation stabilizes tau by inhibiting autophagy-mediated tau degradation. **a**, IB image showing the changes in ubiquitination of tau441 and tau441-Y29F by TYK2 or TYK2KD. **b**, IB image of the K48-linked or K63-linked Ub-tau in the presence of TYK2 showing that TYK2 enhances K63-linked ubiquitination of tau protein. **c**, IB images showing the changes in tau ubiquitination by TYK2 in HEK293T cells treated with either proteasome inhibitor (MG132, 10 μM, 6 h) or autophagy inhibitor (CQ, 40 μM, 6 h). To enhance tau ubiquitination, CHIP and GSK3β were transiently expressed. **d**, IB image and quantification of tau protein level in SHSY-5Y cells treated with TYK2 inhibitor for 48 h in the presence or absence of either proteasome inhibitor (MG132, 100 nM) or autophagy inhibitor (CQ, 20 μM; *n* = 6–11, *n* is the number of biological repeats; one-way ANOVA/Dunnett’s multiple comparison test, *P*_(DMSO–DMSO versus DMSO–TYK2 inhibitor)_ = 0.0003, *P*_(MG132–DMSO versus MG132–TYK2 inhibitor)_ < 0.0001, *P*_(CQ–DMSO versus CQ–TYK2 inhibitor)_ > 0.9999). Data are represented as mean ± s.e.m., ***P < 0.0005 and ****P < 0.0001. **a**–**c**, the representative images of three independent experiments. CQ, chloroquine; CHIP, C-terminus of Hsc70-interacting protein; NS, not significant. Source data

To investigate how TYK2 stabilizes tau protein, we examined whether it regulated tau ubiquitination. In a cellular ubiquitination assay, both TYK2 and TYK2 kinase domain (TYK2KD), which has higher phosphorylating activity^36^, increased the polyubiquitinated tau (Ub-tau) WT but not tau-containing phosphorylation insensitive mutation at Try29 (tau441-Y29F), indicating that the increased Ub-tau by TYK2 was Tyr29 phosphorylation-dependent (Fig. 3a). We reasoned that TYK2 stabilizes particularly Ub-tau protein, leading to accumulation of the Ub-tau protein. TYK2 led to a significant accumulation of K63-linked Ub-tau, which is the second most common form of tau ubiquitination and induces protein degradation predominantly in the lysosome^37,38^. TYK2 showed a minimal effect on K48-linked Ub-tau, which has been implicated in proteasomal degradation (Fig. 3b)^39^. Inhibition of autophagy but not inhibition of proteasome activity abolished the increase in ubiquitinated tau by TYK2 (Fig. 3c). Notably, we also found that the autophagy inhibitor but not the proteasome inhibitor abolished the reduction of tau protein level by the TYK2 inhibitor (Fig. 3d). Collectively, these data demonstrate that TYK2-mediated Tyr29 phosphorylation stabilizes the ubiquitinated tau protein by inhibiting autophagy-dependent tau degradation.

### TYK2 protein level correlates with pathological phospho-tau in human AD brain tissue

**Fig. 4:**
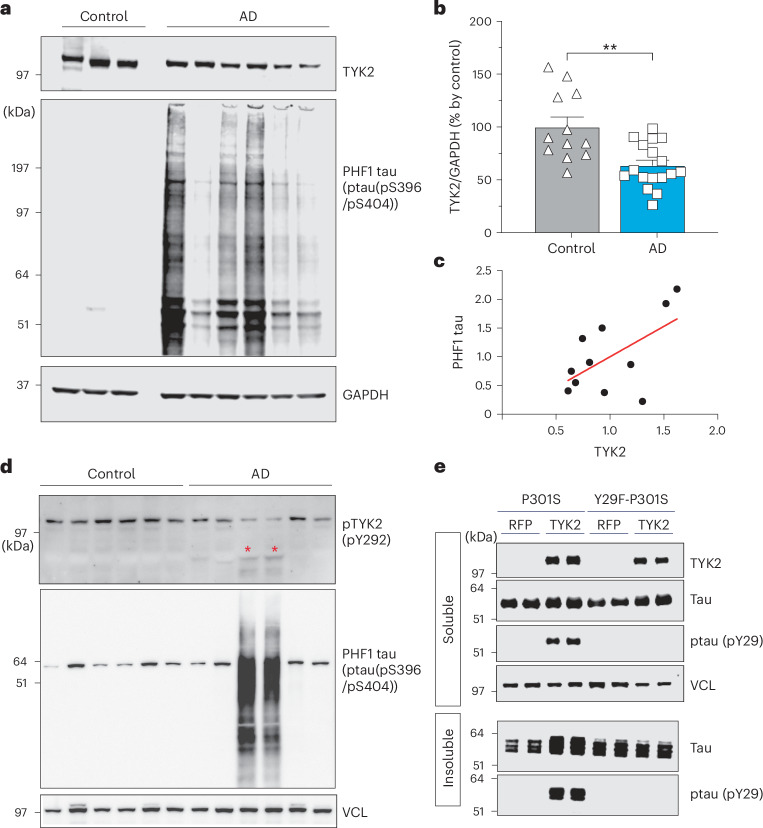
The fragment of TYK2 correlates with pathological phospho-tau in human AD brain samples. **a**,**b**, IB image (**a**) and quantification (**b**) of TYK2 and PHF1 tau (ptau(pS396/pS404)) in human patients with AD and control participants showing TYK2 is reduced in patients with AD compared to control participants (*n* = 12 for control and *n* = 17 for AD, *n* is the number of individuals; unpaired *t* test/two-tailed, *P* = 0.0011). **c**, Graph of individual values (black dots) and trendline (red line) of the level of TYK2 versus the level of PHF1 in AD samples, showing their correlation (slope = 1.06, *P* value = 0.06, *R*^2^ = 0.34). **d**, IB image of the activated TYK2 and PHF1 tau (ptau(pS396/pS404)) in human patients with AD and control participants. Two AD samples with the cleaved TYK2 (red asterisk) had high levels of PHF1 tau. **e**, Representative IB image (four independent repeats) of total tau and PHF1 tau from detergent-soluble or detergent-insoluble fractions from HEK293T cells expressing tau441-P301S or tau441-Y29F/P301S, showing the rise in tau aggregates upon tau seeding in the presence of TYK2 but not when tau441-P301S/Y29F was expressed. Data are represented as mean ± s.e.m., **P < 0.01. Source data

To investigate the potential role of TYK2 in disease progression and tau accumulation, we measured the total TYK2, the active form of TYK2 (pTYK2 at Tyr292)^40^, and pathological tau species in brain tissue samples from patients with AD (Supplementary Table 1) using IB analysis. Patients with AD have reduced TYK2 compared to control human participants (Fig. 4a,b). Notably, there is a trend of positive relationship between TYK2 level and pathological hyperphosphorylated tau (PHF1 tau; Fig. 4c). A subset of AD samples exhibited fragments representative of TYK2 that correlated with hyperphosphorylated tau (Fig. 4d). We hypothesized that Tyr29 phosphorylation by TYK2 promotes the accumulation of pathological tau species. To test this, we expressed a typical pathological mutant tau, tau441-P301S containing either the WT Y29 or the mutated Y29F (tau441-Y29F/P301S), in the presence of TYK2 in HEK293T cells and then separated the detergent-insoluble tau fractions from cell lysate. IB analysis revealed that upon the addition of proteopathic tau seeds (insoluble lysate from older PS19 mice), TYK2 promoted aggregation of tau441-P301S but not tau441-Y29F/P301S (Fig. 4e).

### TYK2 promotes tau aggregation in human cells

**Fig. 5:**
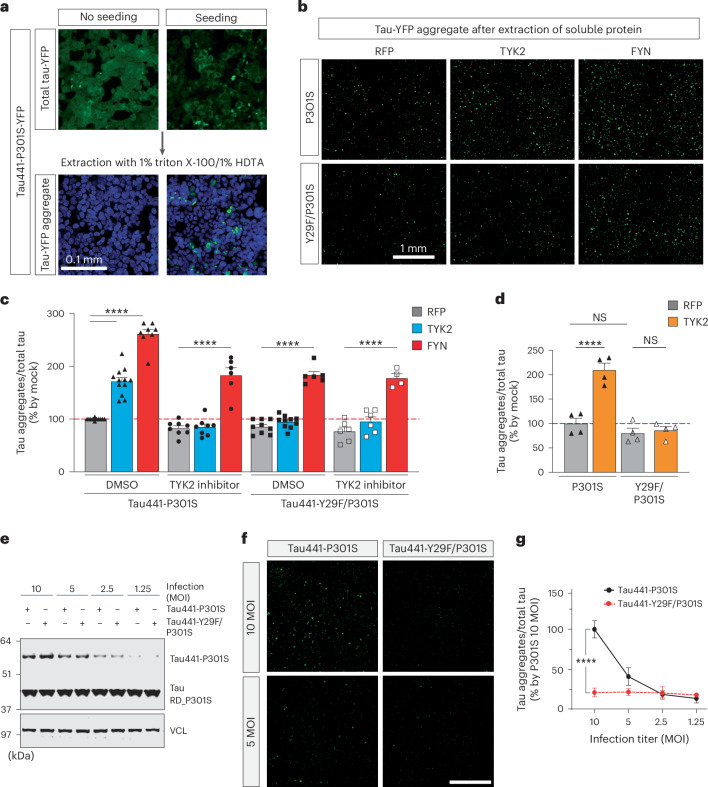
TYK2 promotes tau aggregation upon proteopathic tau seeding in human cells. **a**, Representative image of total tau-YFP (top) and the remaining tau-YFP aggregates (bottom) after extraction of soluble tau protein by the given detergent without or with tau seed transduction, respectively, in HEK293T cells that stably express tau441-P301S-YFP. Scale bar, 0.1 mm. **b**, Representative image of scanned tau-YFP aggregates of indicated samples after 48 h of tau seed transduction. Cells transiently expressed either RFP, TYK2 or FYN (the sample images are from the experiment using DMSO). Entire 24-well plates were scanned to detect total tau protein and then scanned again after extraction of soluble proteins to detect aggregated tau. Scale bar, 1 mm. **c**, Quantification of tau aggregation level from tau441-P301S-YFP and tau441-Y29F/P301S-YFP cells treated with either DMSO or deucravacitinib (TYK2 inhibitor, 100 nM) followed by transfection of either RFP, TYK2 or FYN. TYK2 promoted tau aggregation but not in tau441-Y29F/P301S. This increase was abolished by TYK2 inhibitor, whereas FYN promoted tau aggregation regardless (*n* = 8–12: respective experiments; *n* = 6–10; two-way ANOVA/Tukey’s multiple comparison test, *P*_(tau441-P301S:RFP–DMSO versus TYK2–DMSO)_ < 0.0001, *P*_(tau441-P301S:RFP–DMSO versus FYN–DMSO)_ < 0.0001, *P*_(tau441-P301S:RFP–TYK2 inhibitor versus TYK2–TYK2 inhibitor)_ > 0.9999, *P*_(tau441-P301S:RFP–TYK2 inhibitor versus FYN–TYK2 inhibitor)_ < 0.0001, *P*_(tau441-Y29F/P301S:RFP–DMSO versus TYK2–DMSO)_ = 0.99951, *P*_(tau441-Y29F/P301S:RFP–DMSO versus FYN–DMSO)_ < 0.0001, *P*_(tau441-Y29F/P301S:RFP–TYK2 inhibitor versus TYK2–TYK2 inhibitor)_ = 0.9997, *P*_(tau441-Y29F/P301S:RFP–TYK2 inhibitor versus FYN–TYK2 inhibitor)_ < 0.0001). **d**, Quantification of tau aggregation level from tau biosensor cell line stably expressing tau441-P301S or tau441-Y29F/P301S with either RFP or TYK2 after proteopathic tau seed transduction (*n* = 4; one-way ANOVA/Tukey’s multiple comparison test, *P*_(P301S–RFP versus P301S–TYK2)_ < 0.0001, *P*_(Y29F/P301S–RFP versus Y29F/P301S–TYK2)_ = 0.9811, *P*_(P301S–RFP versus Y29F/P301S–RFP)_ = 0.5897). **e**–**g**, The spontaneous tau aggregation in tau RD P301S FRET biosensor cells after infection with tau441-P301S or tau441-Y29F/P301S. **e**, IB image showing comparable total tau level between tau441-P301S and tau441-Y29F/P301S in the same infection MOI. Representative images (**f**) and quantification graph (**g**) showing the spontaneous tau aggregation after 6 days of tau infection (*n* = 4; two-way ANOVA/Sidak’s multiple comparisons, *P*_(tau441-P301S–10 MOI versus tau441-Y29F/P301S–10 MOI)_ < 0.0001, *P*_(tau441-P301S–10 MOI versus tau441-P301S–5 MOI)_ = 0.0001). Scale bar, 1 mm. *n* is the number of biological repeats. Data are represented as mean ± s.e.m., ****P < 0.0001. Source data

To further investigate the contribution of TYK2-mediated Tyr29 phosphorylation to the accumulation of pathological tau species, we established an assay in which cells overexpress yellow fluorescent protein (YFP)-fused human tau441 containing a proteopathic mutation (P301S) with or without a nonphosphorylatable mutation at Tyr29 (tau441-P301S-YFP or tau441-Y29F/P301S-YFP). Upon tau seed transduction, these transgenic cell lines form aggregates that can be readily measured by the YFP signal after extraction of soluble tau protein (Fig. 5a)^41^. Before seed transduction, cells were treated with TYK2 inhibitor (deucravacitinib) or DMSO and then transfected with either red fluorescent protein (RFP) or TYK2. As a positive control, we transfected cells with FYN, which phosphorylates tau at Tyr18 residue and promotes tau aggregation in a tauopathy mouse model^19^.

In this cellular assay, TYK2 expression increased tau aggregation, but this increase was ablated by either a TYK2 inhibitor (deucravacitinib) or the Y29F mutation. FYN-mediated tau aggregation was moderately decreased by both the TYK2 inhibitor and the Y29F mutation. However, neither condition completely prevented FYN-mediated aggregation compared to basal levels seen in the RFP control, suggesting FYN-induced tau aggregation is independent of TYK2 effects. The moderate reduction could have resulted from either inhibition of endogenous TYK2 or the partial lowering of total tau due to the Y29F mutation (Fig. 5b,c). We reproduced this finding in a different cellular model by virally expressing P301S tau or Y29F/P301S tau in tau biosensor cells (tau RD P301S Förster resonance energy transfer (FRET) biosensor cell) expressing a fluorescence-conjugated microtubule-binding domain—upon tau seeding, TYK2 accelerated the formation of detergent-resistant tau aggregates with P301S cells but not with Y29F/P301S tau (Fig. 5d). Interestingly, these two cellular models revealed tau aggregation by itself without seed transduction, whereas Y29F/P301S tau formed dramatically less tau aggregation (Fig. 5e–g). Together, these data demonstrate that tau phosphorylation at Tyr29 by TYK2 stabilizes tau and facilitates proteopathic tau aggregation in human cells.

### Tau phosphorylation at Tyr29 residue enhances tau nitration at the same residue

**Fig. 6:**
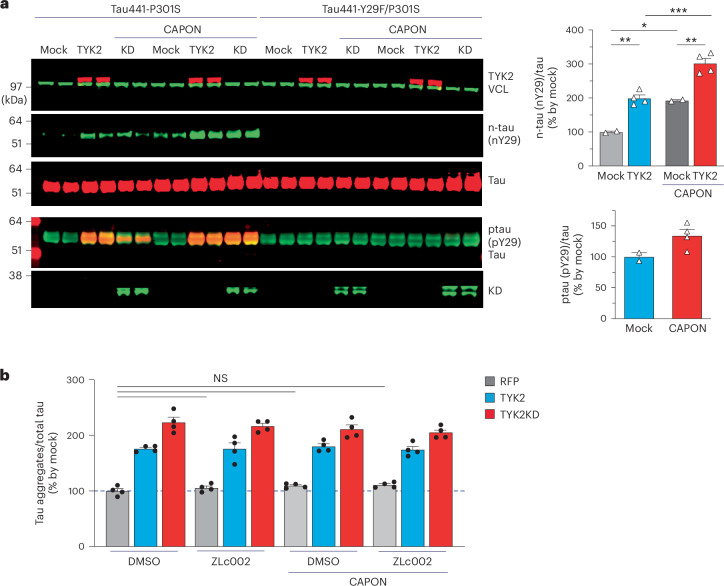
TYK2-mediated tau phosphorylation at Tyr29 augments tau nitration at Tyr29 in HEK293T cells. **a**, IB image and quantification of tau phosphorylation and nitration at Tyr29 residue of WT tau441 in the presence of TYK2 or/and CAPON in HEK293T cells. Tau nitration at Tyr29 residue is increased by phosphorylation at the same residue by TYK2 regardless of CAPON expression (*n* = 4; top-right, one-way ANOVA/Tukey’s multiple comparison, *P*_(Mock versus TYK2)_ = 0.00334, *P*_(CAPON–Mock versus CAPON–TYK2)_ = 0.001830, *P*_(Mock versus CAPON–Mock)_ = 0.011628; bottom-right, unpaired *t* test/two-tailed, *P*_(Mock versus CAPON)_ = 0.0921). **b**, Quantitative graph of tau aggregation from cells treated with DMSO or Zlc002 (CAPON inhibitor, 1 mM), followed by transfection (RFP, TYK2, TYK2KD or/and CAPON) and tau seed transduction (*n* = 4; two-way ANOVA/Tukey’s multiple comparison test, *P*_(DMSO versus ZLc002)_ > 0.9999, *P*_(DMSO versus CAPON–DMSO)_ > 0.9999, *P*_(DMSO versus CAPON–ZLc002)_ = 0.9999, *P*_(CAPON–DMSO versus CAPON–ZLc002)_ = 0.9998). Tau aggregation was increased by TYK2 but was not changed by either the CAPON or Zlc002 (*n* = 4). *n* is the number of biological repeats. Data are represented as mean ± s.e.m., *P < 0.05, **P < 0.01 and ***P < 0.0005. Source data

Tyr29 can undergo nitration as well as phosphorylation^29,30^. The nitrated Tyr29 accumulates in tissues of patients with tauopathy, and CAPON-mediated nitration of Tyr29 leads to the accumulation of pathological forms of tau in a tauopathy mouse model^31,42^. We therefore hypothesized that TYK2 phosphorylation at Tyr29 affects nitration at the same residue, influencing the transformation of native tau species into pathological species. To test this hypothesis, we first transfected HEK293T cells with CAPON, TYK2 or both and examined the nitration and phosphorylation of tau at Tyr29. TYK2-mediated Tyr29 phosphorylation enhanced nitration at Tyr29 as detected by a nitrated Tyr29-specific antibody (Fig. 6a). Next, we examined whether nitration at Tyr29 facilitates tau aggregation in a cellular model by transiently expressing TYK2, CAPON or both. Before transfection, cells were treated with a CAPON inhibitor (Zlc002). Neither overexpression nor inhibition of CAPON affected tau aggregation. We found no significant difference in tau aggregates in the three conditions (Fig. 6b). These data suggest that CAPON nitration of Tyr29 has no effect on tau aggregate formation and that Tyr29 phosphorylation by TYK2 increases the accumulation of tau aggregates regardless of nitration in the in vitro cellular model. We cannot rule out, however, that nitration might need in vivo conditions to manifest its effect on the propagation of tau pathology.

### TYK2-mediated Tyr29 phosphorylation leads to the accumulation of pathological tau species in P301S tau-transgenic mice

**Fig. 7:**
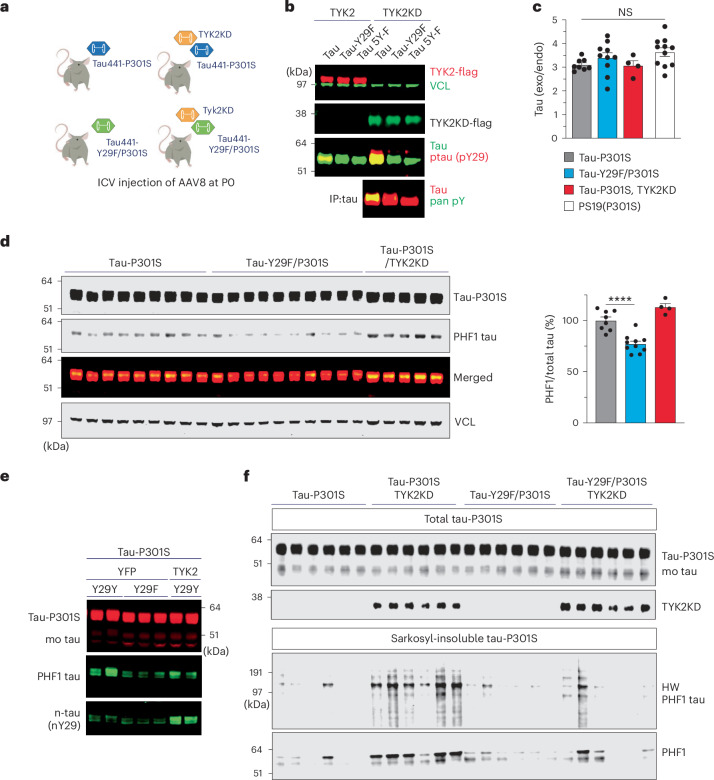
Preventing Tyr29 phosphorylation attenuates tau pathology, but TYK2 expression aggravates pathology in P301S mice. **a**, Experimental design showing transgenic mice that express P301S tau with or without phosphorylation-disable mutation tau (Y29F). Mice were co-injected at P0 with YFP or TYK2KD, and brain tissue was collected for biochemistry at 1 month (**d**) or 2 months (**e** and **f**) after birth. **b**, Representative IB image (three independent repeats) showing the phosphorylation of either tau441, tau441-Y29F or tau441 with nonphosphorylatable mutation at all five Tyr residues (tau5Y-F) by either TYK2 or TYK2KD. TYK2KD phosphorylated mostly Tyr29 indicated by the depletion of pan-phospho-antibody signal. **c**, Relative expression of exogenous (exo) human P301S tau compared to the endogenous (endo) mouse tau from the virus-injected mice or P301S tau-transgenic mice (PS19 line; *n* = 4–10; one-way ANOVA/Tukey’s multiple comparison test, *P*_(P301S versus Y29F/P301S)_ = 0.6720, *P*_(P301S versus P301S–TYK2KD)_ = 0.9996, *P*_(P301S versus PS19)_ = 0.1767). **d**, IB image and quantification graph of total tau and a pathological PHF1 tau from the injected virus (*n* = 4–9; one-way ANOVA/Tukey’s multiple comparison test, *P*_(P301S versus Y29F/P301S)_ = 0.00008, *P*_(P301S versus P301S–TYK2KD)_ = 0.0609). **e**, Representative IB image of total tau, a PHF1 tau and nitrate tau at Tyr29 from mouse brains 1 month after birth (three independent repeats). **f**, Representative IB image of the total, sarkosyl-insoluble or sarkosyl-insoluble HW P301S, indicating that TYK2 enhanced detergent-insoluble tau441-P301S but not tau441-Y29F/P301S in mouse brains 2 months after birth (*n* = 6, two independent repeats). *n* is the number of mice. Data are represented as mean ± s.e.m., ****P < 0.0001. HW, high-molecular-weighted. Source data

To investigate the role of TYK2 in tau phosphorylation in vivo, we generated a new mouse model by delivering AAV8 harboring either tau441-P301S or tau441-Y29F/P301S via P0-ICV injection^43^. Mice were co-injected with YFP or the TYK2KD (Fig. 7a). TYK2KD is a small C-terminal kinase domain that can fit into AAV because it lacks N-terminal regulatory and pseudokinase domains^36^. This truncated TYK2 has greater enzymatic activity than full-length TYK2 (ref. ^36^) but still phosphorylates mainly Tyr29 (Fig. 7b). One or two months after injection, we assessed pathological forms of tau in brain tissue.

**Extended Data Fig. 4:**
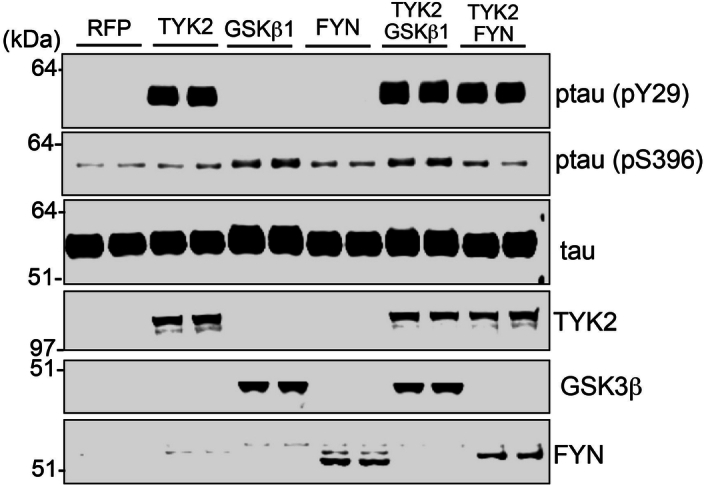
Tau phosphorylation by FYN, TYK2 or/and GSK3β. Representative IB image of the overexpressed total tau and phosphorylated tau at Tyr29, or Ser396 by FYN, TYK2, GSK3β, TYK2/GSK3β or TYK2/FYN, respectively (3 independent experiments). The kinases were transiently transfected and expressed for 48 h in HEK293T cells which stably expressed tau441 WT. Source data

Our tau-transgenic mice expressed three to five times more P301S tau than the endogenous mouse protein, much like the P301S tau-transgenic mouse model (PS19; Fig. 7c)^44^. Similar to the PS19 mice, our virally induced transgenic mice formed PHF1 tau (tau that is pathologically hyperphosphorylated at serine 396 and serine 404) 1 month after birth. P301S tau containing the nonphosphorylatable Y29F developed significantly less PHF1 signal. We also found that P301S tau mice with TYK2KD tended to display more PHF1 signals (Fig. 7d) and a higher nitration signal at Tyr29 (Fig. 7e). Given that PHF1 tau was increased by TYK2-mediated Tyr29 phosphorylation but was decreased in Y29F P301S tau, we tested the interplay of TYK2 with glycogen synthase kinase-3β (GSK3β), which phosphorylates tau at multiple sites including PHF1 sites (Ser396/Ser404)^45–47^ or FYN in cells. TYK2-mediated Tyr29 phosphorylation itself didn’t enhance the GSK3β Ser396 phosphorylation or vice versa (Extended Data Fig. 4), suggesting that Tyr29 phosphorylation stabilizes tau protein and makes it more susceptible to pathogenic associated phosphorylation such as phospho-Ser396/phospho-Thr404 and forming more PHF1.

We next investigated the effect of Tyr29 phosphorylation on tau aggregation by isolating detergent-insoluble tau protein and found that the TYK2KD promoted accumulation of detergent (1% sarkosyl)-insoluble aggregates of P301S tau but not P301S tau containing Y29F. The TYK2KD also increased high-molecular-weight species of insoluble tau protein, which did not develop in P301S with Y29F mutation (Fig. 7f). There was too little aggregation of P301S tau without the TYK2KD to allow analysis of the difference between P301S and Y29F/P301S tau at 2 months of age. Nevertheless, Y29 phosphorylation by TYK2 promotes the accumulation of pathological tau species in a virally induced tauopathy mouse model.

### Knockdown of *Tyk2* alleviates tau pathologies in a tauopathy mouse model

**Fig. 8:**
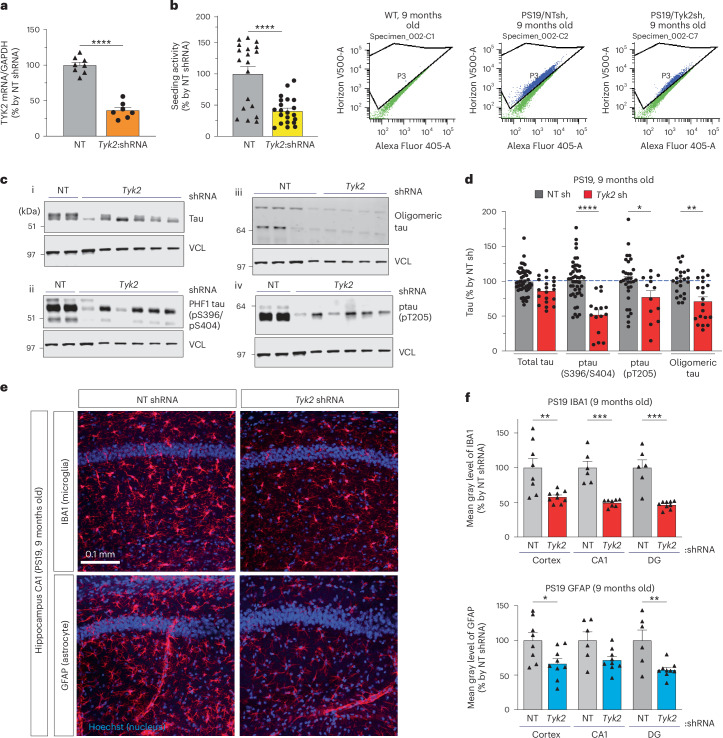
TYK2 knockdown reduces total and pathogenic tau species and attenuates gliosis in P301S tau-transgenic mice (PS19 line). **a**, mRNA level of *Tyk2* from 9-month-old mice ICV-injected with either NT shRNA or *Tyk2* shRNA (*n* = 8; unpaired *t* test/two-tailed, *P* < 0.000001). **b**, In vitro tau aggregation assay to examine proteopathic tau seeding activities. Quantitative graph and representative bivariate plots of tau FRET signal from tau RD P301S FRET biosensor cells after seed transduction with brain lysate from WT and PS19 mice (9 months old). Graph shows the number of FRET-positive cells (*n* = 18–20; unpaired *t* test/two-tailed, *P* < 0.0001). **c**,**d**, IB image (**c**) and quantification (**d**) of total tau (**c**_i, *P* = 0.0049), ptau (pS396/S404) (**c**_ii, *P* < 0.0001), oligomeric tau (**c**_iii, *P =* 0.0001) and ptau (pT205) (**c**_iv, *P* = 0.0147) in PS19 mice after knockdown of *Tyk2* at 9 months of age. Each data point in the bar graphs represents an individual animal (*n* = 15–45; one-way ANOVA/Dunnett’s multiple comparison test). **e**, Representative images of microglia (IBA1) and astrocyte (GFAP) markers in hippocampal CA1 region of 9-month-old PS19/NT and PS19/*Tyk2* knockdown mice. **f**, Quantification of IBA1 intensity (*n* = 6–10; *P*_(cortex–NT versus cortex–Tyk2sh)_ = 0.000418, *P*_(CA1–NT versus CA1–Tyk2sh)_ = 0.000147, *P*_(DG–NT versus DG–Tyk2sh)_ = 0.000044) and GFAP intensity (*n* = 6–10; *P*_(cortex–NT versus cortex–Tyk2sh)_ = 0.022869, *P*_(CA1–NT versus CA1–Tyk2sh)_ = 0.010256, *P*_(DG–NT versus DG–Tyk2sh)_ = 0.006338) in several brain regions of PS19/NT PS19/*Tyk2* knockdown (one-way ANOVA/Sidak’s multiple comparison test) mice. Each data point represents an individual animal, and each animal has an average of two to three sections (*n* = 6–10). *n* is the number of mice. Data are represented as mean ± s.e.m., *P < 0.05, **P < 0.01, ***P < 0.0005 and ****P < 0.0001. Source data

Because we have previously shown that reducing total tau levels by just 15–20% mitigates tau pathology and cognitive impairment in the PS19 mouse model^11,12^, we wanted to see whether partially reducing *Tyk2* function in vivo (~50% reduction) could be a viable therapeutic strategy for decreasing tau levels and mitigating tau pathology. We focused on total tau and pathological tau species as well as gliosis. Using bilateral ICV injection of AAV8 containing *Tyk2* shRNA at P0, we knocked down *Tyk2* in PS19 mice that express human mutant tau (1N4R P301S) at three to five times the level of the endogenous mouse protein and recapitulate many features of human tauopathies^44^. The injected PS19 and WT littermate mice were then aged until 9 months, long enough to develop tauopathy-associated phenotypes, before we collected the mouse brains for biochemical and immunohistochemical analyses. At 9 months after birth, the *Tyk2* shRNA-injected PS19 mice had reduced Tyk2 levels (~60% reduction) compared to nontarget (NT) shRNA-injected PS19 mice (Fig. 8a). PS19 mice display tau seeding activity, which means misfolded tau present in the brain homogenate promotes tau aggregation via a prion-like mechanism^48,49^. To measure tau seeding activity from *Tyk2* knockdown mice, we performed seed transduction using the tau biosensor cells (tau RD P301S FRET biosensor cell) that emit a FRET signal upon tau aggregation^48^. Brain homogenates from PS19 mice with *Tyk2* shRNA showed significantly less proteopathic tau seeding than PS19 control mice, as indicated by fewer FRET-positive cells (Fig. 8b).

We then examined the levels of different tau species in brain lysates from these mice. At 9 months of age, PS19/*Tyk2* knockdown mice had markedly lower levels of total tau and pathological forms of tau (pT205, pS396/S404 residues and oligomeric tau; Fig. 8c,d). PS19 mice with *Tyk2* knockdown also had much less microgliosis and astrogliosis than PS19 control mice, as indicated by reduced ionized calcium‐binding adaptor molecule 1 (IBA1) and glial fibrillary acidic protein (GFAP) staining, respectively, in the cortex, the CA1 area of the hippocampus and the dentate gyrus (DG) (Fig. 8e,f).

## Discussion

Growing evidence suggests that post-translational modification has a critical role in regulating normal function and pathological features of tau in disease conditions. Regarding the pathological properties of tau, only the phosphorylation of serine and threonine has been well studied, while tyrosine phosphorylation has only recently been described. These first studies, however, demonstrated the importance of tau phosphorylation by tyrosine kinases in the pathogenesis of tauopathies including AD^17,19,50–52^. For instance, the partial reduction in phosphorylation of tau at Tyr18 leads to remarkably reduced tau aggregation (NFTs) in FYN knockout tauopathy mice (rTg4510/FYN^−/−^)^19^. Interestingly, tau phosphorylation on Tyr29 has been described in human patients with AD previously by mass spectrometry analysis^3^, but its pathological consequences are not known.

Here we show that TYK2 and its phosphorylation of tau at Tyr29 residue contribute to tau pathology by directly affecting tau concentrations in human cells and the mouse brain. A previous study suggested that TYK2/STAT3 signaling is involved in AD pathophysiology, such that Aβ-induced activation of TYK2 leads to caspase 3-mediated apoptotic cell death in primary neuronal cultures^53^. Together with our findings, these data suggest that TYK2 might be downstream of Aβ-induced pathology and upstream of tau pathology, making it a candidate contributor to the toxic effects of Aβ on tau in AD, in addition to AMP-activated protein kinase^54–56^, GSK3β^57^ and others^58^.

The Holtzman group has identified antitau monoclonal antibodies, including intrabody HJ8.5, that can destabilize tau and block its seeding activity, mitigating the cognitive deficits in a tauopathy mouse model^33^. This indicates that the HJ8.5 epitope region of tau, which spans Tyr29, has an important role in tau-associated pathogenesis^33,59,60^. Supporting these previous studies, our results show that TYK2 stabilizes tau via phosphorylation at Tyr29 by inhibiting autophagic tau degradation and promoting the accumulation of pathogenic tau species. Collectively, our findings and the Holtzman lab’s previous work demonstrate that TYK2 is critical to prevent tau aggregation. It will be important to better understand TYK2 to ensure that it is a safe therapeutic target; for example, we know that *Tyk2* knockout mice exhibit a mild immunodeficiency^61^ and that humans carrying two loss-of-function alleles and thus completely lacking this enzyme develop immunodeficiency 35 (ref. ^62^). The fact that the heterozygous parents of these individuals are healthy, however, is encouraging, and partial reduction of TYK2 via viral infection of *Tyk2* shRNA was sufficient to reduce tau levels and mitigate astrogliosis and microgliosis seen in tauopathy mouse models.

In addition to phosphorylation, the Tyr29 residue of tau can be nitrated, and this nitration is closely related to pathology. The binder group showed that nitrated Tyr29 (tau-n29) labeled the fibrillar lesions of tauopathy brains, and this nitration markedly altered the ability of tau to self-associate and promote tubulin assembly^31,63^. Our study found that tau-pTyr29 by TYK2 enriched the nitration of tau at the same site, tau-nTyr29. But neither tau-nTyr29 by itself nor tau-pTyr29/Tyr29 facilitated tau aggregation compared to control (RFP or tau-pTyr29 only) in vitro. It is noteworthy that the appearance of the tau-nTyr29 occurs in a subset of tau inclusions and primarily occupies the mature, compact fibrillar tangle^31^. It is likely that the promotion of tau aggregation by tau-nY29 modification does not alter acute tau aggregation in vitro and requires the in vivo context to facilitate progression to more mature NFTs.

In summary, TYK2 phosphorylates tau at Tyr29, leading to its accumulation. Partial reduction of Tyk2 postnatally reverses tau-associated pathological phenotypes in two different tauopathy mouse models. The modest suppression of tau levels by reducing TYK2 can mitigate tau-associated pathological phenotypes even in a tau-transgenic mouse model. Collectively, these data suggest that partial TYK2 suppression is a potential strategy to reduce tau toxicity.

## Methods

Baylor College of Medicine (BCM) Institutional Animal Care and Use Committee (IACUC) approved all mouse care and manipulation. Mice were housed in an American Association for Laboratory Animal Science-certified level three facility. All mice were maintained in a 14 h light/10 h dark cycle at 68–72 °F and 30–70% humidity, with standard mouse chow and water ad libitum. Mice were monitored daily by veterinary staff. All procedures to maintain and use these mice were reviewed and approved animal protocol (AN-1013) by the BCM IACUC in accordance with the guidelines of the US National Institutes of Health (NIH).

### Materials

The materials used in the present study are given in Supplementary Table 2.

#### Mouse models

CFW mice were used for in vivo validation of *Tyk2*. Timed pregnant Swiss Webster (CFW) mice or FVB mice were used for embryonic primary cortical neuronal culture experiments. Tau P301S transgenic mice (PS19 line, (C57BL/6 x C3H) F1) were bred in our laboratory. *Tyk2* knockdown mice in PS19 background were generated by ICV injection of the recombinant AAV8 harboring *Tyk2* shRNA. Transgenic offspring for these experiments were generated by mating PS19 males with FVB females. Pathogenic tau mice (P301S tau 1N4R) were generated by ICV injection of AAV harboring tau441-P301S or tau441-Y29F/P301S with/without human TYK2KD in FVB background.

#### Antibody dilution

The following primary antibodies were used: anti-TYK2 (Abcam, ab303500; 1:1,000); antiphospho-TYK2 (p292; Abcam, ab138394; 1:1,000); PHF1 (Peter Davies, 1:2,000); antitau (Abcam, ab80579; 1:4,000); antitau (Agilent, A0024; 1:10,000); anti-HA (BioLegend, 901514; 1:4,000); anti-Flag (M2; Sigma-Aldrich, F3156, 1:4,000); anti-ptau (pT205; Thermo Fisher Scientific, 44-738G; 1:2,000); antitau, oliogomeric (Merk Millipore, ABN454-I; 1:1,000); antiphospho-Tyr (Merk Millipore, 05-321; 1:1,000); anti-FYN (Cell Signaling Technology, 4023; 1:1,000); antivinculin (Sigma-Aldrich, V9131; 1:2,0000); anti-GAPDH (ImmunoChemical, 2-RGM2; 1:20,000); anti-GFAP (Norus Biologicals, 53809; 1:1,000); anti-IBA1 (Wako, 019-19741; 1:1,000); antiphospho-tau (pTyr29; not available, 1:1,000); antiphospho-tau (pTyr18; MediaMabs, MM-0194-P; 1:1,000); antiphopsho-tau (pS396); PHF13 (Cell Signaling Technology, 9632S; 1:1,000); anti-DUSP (ABclonal, A2919; 1:1,000) and anti-β-catenin (Cell Signaling Technology, 8480; 1:1,000).

The following secondary antibodies were used: donkey antirabbit IgG, Alexa Fluor 555 (Thermo Fisher Scientific, A-31572; 1:500); donkey antigoat IgG, Alexa Fluor 594 (Jackson ImmunoResearch, 705-585-003; 1:500); donkey antirabbit IgG, IRDye 800CW (Li-COR Bioscience, 926-32213; 1:10,000); goat antirabbit IgG, IRDye 680RD (LI-COR Bioscience, 926-68071; 1:10,000); goat antimouse IgG, IRDye 800CW (LI-COR Bioscience, 926-32210; 1:10,000) and goat antimouse IgG, IRDye 680RD (Li-COR Bioscience, 926-68072; 1:10,000).

### Method details

#### Cloning

We designed four to five individual shRNA sequences per target gene, using the SplashRNA algorithm^64^. The shRNA sequence was amplified by PCR, using one pair of oligomers set (miRE-Gib-forward, 5′-ttcttaacccaacagaaggctcgagaaggtatattgctgttgacagtgagcg; miRE-Gib-reverse, 5′-gtaaacaagataattgctcgaattctagccccttgaagtccgaggcagtaggca), followed by integration into pAAV-YFP-miRE vector through Gibson cloning as previously described^65^.

Precision LentiORFs are human cDNA open reading frames (ORFs) cloned into a lentiviral expression vector to overexpress the given human proteins in mammalian cells.

Human TYK2 and TYK2KD, containing flag tag at the C-terminal, were amplified by PCR and cloned into the linearized pLenti-EF1a or pAAV-chicken β actin promotor cut by BamH1/EcoR1 or EcoR1/Not1 using a Gibson Assembly reaction. For tau441 WT containing mutation (Y18F, Y29F, Y197F, Y310F or/and Y397F), tau441-P301S or tau441-Y29F/P301S, two split DNA fragments were amplified, introducing mutation by mutagenic primer, and were joined by assembly reaction to generate full-length tau cDNA. The primers used are given in Supplementary Table 3.

#### Viral production

Recombinant virus containing shRNA or ORF was produced in low-passage HEK293T cells via triple transfection at 80–90% confluency, using TransIT-293 transfection reagent according to the manufacturer’s instructions. For lentiviral packing, cells were transfected with viral shuttle vector (pGIPz, pLenti and pLOC), psPAX2 and pMD2.G at a 4:3:1 ratio. The medium was collected at 48 and 72 h post-transfection. Lentivirus was concentrated 50-fold using a Lenti-X concentrator (Clontech, 631231) in case a higher titer and more purified viruses were required. Recombinant AAV8 was produced using a triple transfection with pAAV, capsid and helper plasmid in a 150 mm dish format, and the resultant virus was purified and concentrated on an iodixanol step gradient, as previously described^66,67^. To increase the yield of virus particles, cell-associated and medium-containing secreted AAVs were collected separately at 72 h post-transfection and combined before purification.

#### Cell culture, transfection, infection and drug treatment

All cell lines were cultured according to the manufacturer’s protocol. Cells were transfected with plasmids encoding cDNA or shRNA using Lipofectamine 2000 or TransIT-293 transfection reagent, according to the manufacturer’s protocol, and incubated for 48–72 h followed by the designed experiments. Cells were infected with lentivirus expressing ORF at 3–10 multiplicity of infection (MOI) in the various cell culture size formats. After 24–48 h of infection, cells were incubated with the appropriate antibiotics at least for 72 h. Cells were further maintained until additional treatment or collection.

Cortical neurons isolated from E16.5 FVB embryos were plated at a density of ~300,000 per well on poly-d-lysine (0.1 mg ml^−1^)-coated 24-well plates. Neurons were maintained in NB-B27 medium (neurobasal plus medium + 1X B27 supplement, 0.5 mM glutamax and 0.2× penicillin–streptomycin; Invitrogen). At day in vitro 3 (DIV3), neurons were infected with recombinant lentivirus at 3–5 MOI. At DIV10, neurons were treated with DOX at a concentration of 50 nM to initiate transgene expression for 24 h.

#### ICV injection of AAV at postnatal day 0 (P0-ICV injection of AAV)

The knockdown efficiency of each shRNA in pAAV was tested in Neuro2A cells as previously described^68^. The two most potent shRNAs targeting *Tyk2* or Luciferase gene as NT control were packed into AAV8 (ref. ^68^). AAV8 harboring a shRNA expression cassette was bilaterally infused into the mouse brain at 4–6 × 10^10^ viral genomes per hemisphere using ICV injection at postnatal day 0 as previously described^69^. For protein overexpression in the brain, AAV8 containing cDNA expression cassette injection at 1–2 × 10^10^ viral genomes per hemisphere. For tau pathology, transgenic offspring were generated by mating PS19 males with FVB females.

#### Mouse brain sample preparation

Mice were killed by isoflurane inhalation at 3 weeks or 2 months postinjection. The forebrain region was collected and immediately frozen on dry ice. For pathology, PS19 mice and age-matched WT littermate mice were killed by sodium pentobarbital overdose and transcardially perfused with ice-cold PBS at 9 months postinjection. The whole hemisphere was isolated and fixed by 4% paraformaldehyde (PFA) for 48 h followed by cryopreserving in 1× PBS containing 30% sucrose at 4 °C for histological study. For the biochemical study, cortex and hippocampus from the leftover hemisphere were dissected together and frozen immediately. Frozen samples were homogenized using an electric pestle (handheld polytron; WPR, 47747-370) in 10× volumes/weight of cold resuspension buffer (1× PBS containing 5 mM EDTA, protease inhibitor cocktail and phosphatase inhibitor cocktail). Part of the homogenate was then diluted 1:1 with RIPA buffer (1× PPS containing 5 mM EDTA, protease inhibitor cocktail, phosphatase inhibitor cocktail, 1% deoxycholate, 1% Triton X-100 and 0.5% SDS). After mixing well, the supernatant was collected by centrifugation at 15,500*g* for 20 min for IB analysis. For the preparation of detergent-insoluble tau, brain homogenate removed tissue debris by centrifugation (2,000*g* for 5 min at 4 °C) was mixed with the same volume of 2× concentrated RIPA buffer without SDS and was centrifuged at 100,000*g* for 30 min at 4 °C. The supernatant was collected (S1), and the pellet was dissolved by incubation with 1× RIPA containing 1% sarkosyl (without SDS) for 1 h at room temperature on an orbital shaker (P1). The soluble fraction (S2) was collected by centrifugation at 200,000*g* for 30 min at 4 °C. Pellet was dissolved in 1× PBS by sonication (probe sonicator, 30% amplitude, pulsed ten times with 2′/1′ on/off; P2). Each sample was mixed with protein-loading dye and boiled at 95 °C for 5 min. RNA was further purified from homogenate using the RNeasy mini kit (Qiagen) as needed.

#### Human tissue sample preparation and IB analysis

Postmortem brain tissues from participants with AD and control participants were provided in the form of frozen blocks by the Massachusetts Alzheimer’s Disease Research Center, which has Institutional Review Board (IRB) approval for the tissue bank and for sharing deidentified autopsy samples (IRB 1999P009556). Specifically, frozen tissues from the middle temporal cortex of patients with AD (*n* = 17) and control individuals (*n* = 12) were obtained (Supplementary Table 1). Tissue donor anonymity was assured by the Massachusetts Alzheimer’s Disease Research Center. AD cases consisted of pathologically severe AD, stages V–VI. Each brain was homogenized in RIPA buffer with a protease inhibitor cocktail (Roche) and diluted in RIPA to 1:10 (wt/vol). Samples were then centrifuged at 16,363*g* for 15 min at 4 °C. The supernatants were portioned into aliquots, snap-frozen and stored at 80 °C until analyzed. Samples were mixed with running buffer, run on a gel and analyzed by IB. Primary antibodies used were anti-pTyk2 (pY292; 1:1,000), PHF1 (1:2,000, gift from P. Davies), antivinculin (1:20,000) and GAPDH (1:50,000).

#### Cell lysate preparation

Frozen cells in plates were thawed on ice briefly and were lysed by gentle mixing with ice-cold cell-lysis buffer (50 mM Tris, 150 mM NaCl, 2 mM CaCl_2_, 1% Triton X-100, 5 mM EDTA and 5% glycerol (pH7.5)) containing 1× protease and phosphatase inhibitor cocktails on shaker for 30–60 min at 4 °C. Cell supernatant was collected by centrifugation at 15,000*g* for 30 min and then subjected to further experiments. The total protein concentration of brain tissue and cell lysate was determined with a BCA Protein Assay Kit. For detergent-insoluble tau faction, cell lysate in ice-cold lysis buffer was subjected to spinning down at 1,250*g* for 10 min at 4 °C, and then the supernatant was further centrifuged at 100,000*g* for 30 min at 4 °C. Supernatant was collected as detergent-soluble tau fraction. The pellet was dissolved in 1× PBS by sonication (probe sonicator, 30% amplitude, pulsed ten times with 2′/1′ on/off; P2). Each sample was mixed with protein-loading dye and boiled at 72 °C for 10 min.

#### Immunoprecipitation

Supernatant from one well of a six-well plate format was incubated with 0.75–1 µg of antitau antibody or 1 µg of anti-Flag antibody conjugated with protein G-conjugated Dynabead (15 µl slurry) for 4 h at 4 °C. After washing with lysis buffer four times, immunoprecipitated proteins were eluted in 30 µl of 1.5× Laemmli sample buffer by boiling 75 °C for 10 min.

#### IB analysis

Lysate (1–1.5 μg μl^−1^) in 1× sample buffer was denatured at 75 °C for 10 min. To detect tau from the cultured cells, 10–15 μg of the sample was incubated with antitau antibody (Dako; 1:8,000), whereas 5–10 μg or 2–5 μg of sample from WT or PS19 mouse brain was detected by antitau antibody (1:4,000). Protein samples were resolved on precast 4–12% Bis–Tris gels (3-(N-morpholino)propanesulfonic acid running; Invitrogen) and transferred onto nitrocellulose membranes (Bio-Rad, 1620145) in Tris–glycine buffer supplemented with 10% methanol at 120 mV for 100 min at 4 °C. After blocking 30 min with 5% bovine serum albumin, the membrane was incubated with the indicated primary antibodies overnight at 4 °C. The protein bands were visualized using corresponding fluorescent secondary antibodies. Infrared fluorescence was measured with the Odyssey CLx imager (LI-COR) and quantified using Image Studio software version 5.2 (LI-COR Biosciences).

#### FRET measurement of seeding activity

Tau-seeded transduction of the tau RD P301S FRET biosensor cell line and flow cytometry analysis were conducted as previously described^12^. Lipofectamine diluent consisting of 7.5 μl Opti-MEM (Gibco, 31985070) and 0.1−0.4 μl Lipofectamine 2000 (Invitrogen, 11668-500) mixed with proteopathic seed diluent consisting of 7.5 μl Opti-MEM and 0.5–2.0 μg protein extract from brain homogenate of tauopathy mouse model was incubated for 30 min at room temperature and added to cells in a 96-well plate format with 60–70% confluency. After 24–48 h of seed transduction, cells were dissociated into single cells in 1× PBS containing 2 mM EDTA and 3% fetal bovine serum. Upon excitation by a 405 nm laser, we measured the FRET signal with the BD LSRFortessa cell analyzer. For data collection, BD FACSDiva Software (v9.0) was used. For each experiment, we recorded cyan fluorescent protein (CFP) and FRET-YFP signals of 50,000–80,000 singlet events at 485/22 nm filter and 525/50 nm filter, respectively. In the bivariate plot, to assess the number of FRET-positive cells, we created a gate by using the FRET-negative signal exhibited by biosensor cells treated with Lipofectamine alone and the FRET-positive signal exhibited by HEK293T cells expressing CFP fused with YFP.

#### In vitro tau aggregation assay in a cellular model

To assess in vitro tau aggregation upon extracellular tau seeding, we used sarkosyl-insoluble tau fraction purified from 8- to 10-month-old PS19 mice as seeding material, as previously described (‘Mouse brain sample preparation’). The concentration of tau was determined by the tau ELISA kit.

The stable cell line (293T: tau441-P301S-YFP or 293T: tau441-Y29F/P301S-YFP) as seed-recipient cells were plated at 50,000 cells per well in a 24-well format plate and were reverse-transfected with plasmids containing cDNA of RFP, TYK2, TYK2KD or FYN with or without CAPON, using TransIT-293 transfection reagent according to the manufacturer’s protocol. Twenty-four hours later, seed transduction complexes (mixture of 15 μl Opti-MEM + 0.5 μl Lipofectamine 2000 and 15 μl Opti-MEM + proteopathic seeds) were incubated for 30 min at room temperature and added to cells. At 24–48 h after seed transduction, the cell medium was replaced with 2% Triton X-100 in 1× PBS, and cells were incubated for 1 min at room temperature. Then cells were added with the same volume of 2% hexadecyltrimethylammonium bromide and further incubated for 10 min at room temperature to remove soluble proteins. Cells were then fixed with 4% PFA for 20 min at room temperature. The whole plates were then scanned to capture tau-YFP aggregates using a cell imaging microplate reader (BioTek, Cytation5 Cell Imaging Reader) at ×25 magnification (3,402 × 3,001 pixels, 13.2 × 11.6 μm per field) and analyzed using Fiji software. For image-data acquisition, BioTek Gen6 Data Analysis Software was used. For total tau control, the total YFP signal from each well was captured before the extraction of soluble protein.

#### Immunofluorescence and microscopy assay

Fixed brains were sagittally sectioned (40 µm thickness) using a sliding microtome (Leica, SM2010 R). Floating sections located 1–1.5 mm from the midline were selected for staining and analysis based on landmarks in the hippocampus, lateral ventricle and striatum. A total of three to four sections were imaged per animal. Brain sections were immunostained with GFAP (1:2,000) and IBA1 (1:2,000) antibodies at 4 °C overnight. Sections were then incubated in Alexa Fluor-conjugated goat secondary antibodies corresponding to primary antibodies and Hoechst (1:2,000) at 4 °C overnight. Fluorescence images were collected from nonoverlapping fields within the somatosensory cortex (above CA1), hippocampus CA1 and the dentate gyrus. A single optical plane of 0.977 µm in depth was collected in blue (Hoechst) and red (GFAP or IBA1) channels using fluorescence microscopy (Carl Zeiss) at ×100 magnification (895.26 µm × 670.8 µm per field). For representative images, z-stacked fluorescence images (14 optical images) were collected from the same field of the cortex and hippocampus CA1 region using confocal microscopy (Carl Zeiss) at ×200 magnification (416.80 µm × 416.80 µm). For image-data acquisition, ZEN microscopy software was used. Then the collected image data were analyzed using Fiji software.

#### Data collection and statistical analyses

All statistical analyses were done using GraphPad Prism 9.0. No statistical methods were used to predetermine sample sizes, but our sample sizes are similar to those reported in previous publications^12^. All data collection and analysis were performed blind to the condition of the experiments. For the purpose of data analysis, all data were collected, excluding data from experimental groups where the positive and negative controls did not function correctly. For animal experiments, because our study involved the entire population of mice rather than a sample subset, traditional randomization techniques were not applicable. Our analysis was conducted on the full cohort, ensuring that all data points contributed to the final results. For the scatter dot plot, data distribution was assumed to be normal, but this was not formally tested. For other types of plots, normality and equal variances were tested by the Shapiro–Wilk test. All comparisons between groups were two-sided. Comparisons between the two groups were analyzed using unpaired *t* test. All comparisons involving more than two groups were analyzed using one-way analysis of variance (ANOVA) followed by Bonferroni post hoc tests, Dunnett’s multiple comparison test, Tukey’s multiple comparison test and Sidak’s multiple comparison test as indicated in figure legends. All reported *P* values are for post hoc comparisons. Adjustments were made for all multiple comparisons. All graphs display group mean ± s.e.m. (*P < 0.05, **P < 0.01, ***P < 0.001 and ****P < 0.0001).

#### Inclusion and ethics

We worked to ensure sex balance in the selection of nonhuman participants. The author list is gender balanced, and we worked to achieve gender balance in our reference list.

### Reporting summary

Further information on research design is available in the Nature Portfolio Reporting Summary linked to this article.

## Online content

Any methods, additional references, Nature Portfolio reporting summaries, source data, extended data, supplementary information, acknowledgements, peer review information; details of author contributions and competing interests; and statements of data and code availability are available at 10.1038/s41593-024-01777-2.

## Supplementary information

Supplementary Information Supplementary Fig. 1 (exemplifying the gating strategy of Fig. 8b).

Reporting Summary

Supplementary Table 1 Pathological and clinical information for postmortem human brain samples.

Supplementary Table 2 Key resources table.

Supplementary Table 3 Sequences of oligonucleotides.

## Source data

Source Data Fig. 1 Unprocessed western blots.

Source Data Fig. 2 Unprocessed western blots.

Source Data Fig. 3 Unprocessed western blots.

Source Data Fig. 4 Unprocessed western blots.

Source Data Fig. 5 Unprocessed western blots.

Source Data Fig. 6 Unprocessed western blots.

Source Data Fig. 7 Unprocessed western blots.

Source Data Fig. 8 Unprocessed western blots.

Source Data Extended Data Fig. 1 Unprocessed western blots.

Source Data Extended Data Fig. 2 Unprocessed western blots.

Source Data Extended Data Fig. 3 Unprocessed western blots.

Source Data Extended Data Fig. 4 Unprocessed western blots.

## Data Availability

Any additional information required to reanalyze the data reported in this paper is available from the lead contact upon request. Source data are provided with this paper.

## Code availability

This study did not generate standardized datatypes for public repositories. This paper does not report original code. DOI is listed in the key resources table.
