# Supplementary material for: TYK2 regulates tau levels, phosphorylation and aggregation in a tauopathy mouse model
Source: Nat Neurosci. 2024 Nov 11;27(12):2417–29. doi: 10.1038/s41593-024-01777-2 (PMC11614740; doi:10.1038/s41593-024-01777-2)
Supplement: Supplementary file 1 — Supplementary Fig. 1 (exemplifying the gating strategy of Fig. 8b). [file 41593_2024_1777_MOESM1_ESM.pdf]

nature neuroscience

Supplementary information
https://doi.org/10.1038/s41593-024-01777-2

TYK2 regulates tau levels, phosphorylation and aggregation in a tauopathy mouse model

In the format provided by the authors and unedited

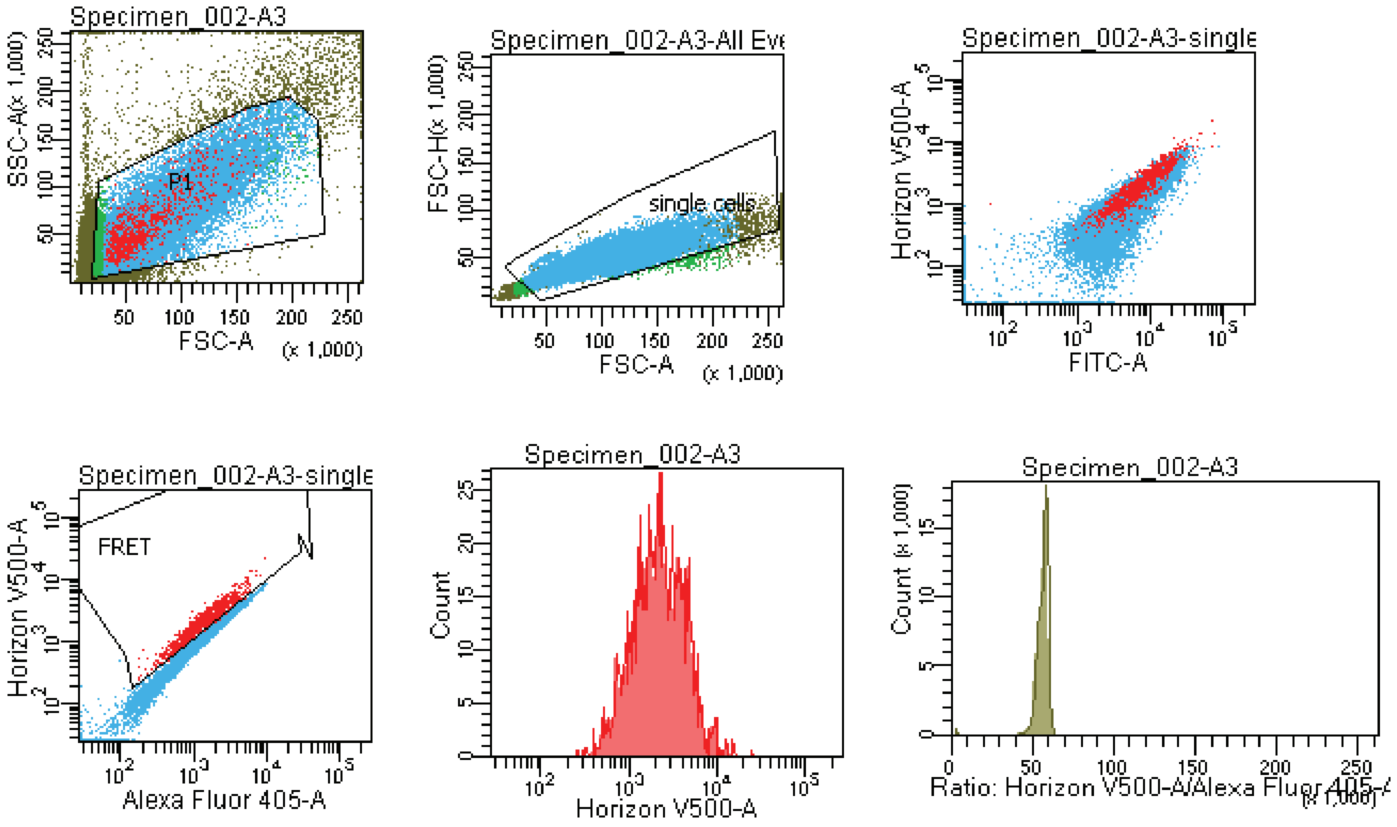

Well: A3

Population	#Events	%Parent	%Total
All Events	122,752	####	100.0
P1	113,927	92.8	92.8
single cells	101,094	88.7	82.4
FRET	996	1.0	0.8

Experiment Name: Ji Tau biosenseor test
Specimen Name: Specimen_002
Well Name: A3
Record Date: Jul 6, 2020 7:12:08 PM
GUID: 972a2783-fa28-42e4-9748-c02a78040bcd

Population	#Events	%Parent	Horizon V500-A Mean	Horizon V500-A Median	Horizon V500-A SD
All Events	122,752	####	1,013	771	1,007
P1	113,927	92.8	996	793	734
single cells	101,094	88.7	1,042	833	749
FRET	996	1.0	2,485	1,999	1,916
