# Supplementary material for: TYK2 regulates tau levels, phosphorylation and aggregation in a tauopathy mouse model
Source: Nat Neurosci. 2024 Nov 11;27(12):2417–29. doi: 10.1038/s41593-024-01777-2 (PMC11614740; doi:10.1038/s41593-024-01777-2)
Supplement: Supplementary file 2 — Reporting Summary [file 41593_2024_1777_MOESM2_ESM.pdf]

nature portfolio

Corresponding author(s): Huda Yahya Zoghbi

Last updated by author(s): Jul 16, 2024

# Reporting Summary

Nature Portfolio wishes to improve the reproducibility of the work that we publish. This form provides structure for consistency and transparency in reporting. For further information on Nature Portfolio policies, see our Editorial Policies and the Editorial Policy Checklist.

## Statistics

For all statistical analyses, confirm that the following items are present in the figure legend, table legend, main text, or Methods section.

| n/a | Confirmed | |
|---|---|---|
| ☐ | ☒ | The exact sample size (*n*) for each experimental group/condition, given as a discrete number and unit of measurement |
| ☐ | ☒ | A statement on whether measurements were taken from distinct samples or whether the same sample was measured repeatedly |
| ☐ | ☒ | The statistical test(s) used AND whether they are one- or two-sided<br>*Only common tests should be described solely by name; describe more complex techniques in the Methods section.* |
| ☒ | ☐ | A description of all covariates tested |
| ☐ | ☒ | A description of any assumptions or corrections, such as tests of normality and adjustment for multiple comparisons |
| ☒ | ☐ | A full description of the statistical parameters including central tendency (e.g. means) or other basic estimates (e.g. regression coefficient) AND variation (e.g. standard deviation) or associated estimates of uncertainty (e.g. confidence intervals) |
| ☐ | ☒ | For null hypothesis testing, the test statistic (e.g. *F*, *t*, *r*) with confidence intervals, effect sizes, degrees of freedom and *P* value noted<br>*Give P values as exact values whenever suitable.* |
| ☒ | ☐ | For Bayesian analysis, information on the choice of priors and Markov chain Monte Carlo settings |
| ☒ | ☐ | For hierarchical and complex designs, identification of the appropriate level for tests and full reporting of outcomes |
| ☒ | ☐ | Estimates of effect sizes (e.g. Cohen's *d*, Pearson's *r*), indicating how they were calculated |

*Our web collection on statistics for biologists contains articles on many of the points above.*

## Software and code

Policy information about availability of computer code

| | |
|---|---|
| Data collection | Image Studio software (Li-COR Biosciences, version 5.2) was used for collecting western blot data.<br>BioTek Gen6 Data Analysis Software  software was used to collect cell images of whole cell plate for the in vitro seeding assay.<br>ZEN Microscopy Software was used to collect images of the immunofluorescence stained brain tissue sections.<br>BD FACSDiva Software v9.0 was used to cellecting cell cytometery data for FRET measurement of seeding activity.<br>SplashRNA algorithm was used to design shRNA sequence for target gene.<br>We didn't use any custom algorithms or software for data collection in this study. |
| Data analysis | Image Studio software (Li-COR Biosciences, version 5.2) was used to quantify the western blots data.<br>Fiji/ImageJ was used to analyze images (In-vitro seeding assay and Immunofluorescence).<br>Data was statistically analyzed using GraphPad Prism (v9.0).<br>We didn't use any custom algorithms or software to analyze data in this study. |

For manuscripts utilizing custom algorithms or software that are central to the research but not yet described in published literature, software must be made available to editors and reviewers. We strongly encourage code deposition in a community repository (e.g. GitHub). See the Nature Portfolio guidelines for submitting code & software for further information.

## Data

Policy information about availability of data

All manuscripts must include a data availability statement. This statement should provide the following information, where applicable:

- Accession codes, unique identifiers, or web links for publicly available datasets
- A description of any restrictions on data availability
- For clinical datasets or third party data, please ensure that the statement adheres to our policy

This study did not generate standardized datatypes for public repositories. We do not have any restrictions on data availability. Data is available upon request. We do not have any clinical dataset or third party data.

No custom code was generated or used in this study

## Research involving human participants, their data, or biological material

Policy information about studies with human participants or human data. See also policy information about sex, gender (identity/presentation), and sexual orientation and race, ethnicity and racism.

| | |
|---|---|
| Reporting on sex and gender | See above and Supplementary Data 1. |
| Reporting on race, ethnicity, or other socially relevant groupings | See above and Supplementary Data 1. |
| Population characteristics | See above and Supplementary Data 1. |
| Recruitment | Brain samples were derived from postmortem tissue of AD patients or normal subjects. Brain biospecimens were provided by the Brain Resource Center at Johns Hopkins and Massachusetts General Hospital. Recruitment details were not specified in this manuscript. We didn't have self-selection or other biases in human biological material in this study. |
| Ethics oversight | No ethics approval was obtained for the present study as postmortem brain tissues from subjects were provided in the form of frozen blocks by the Brain Resource Center at Johns Hopkins and Massachusetts General Hospital. |

Note that full information on the approval of the study protocol must also be provided in the manuscript.

# Field-specific reporting

Please select the one below that is the best fit for your research. If you are not sure, read the appropriate sections before making your selection.

☒ Life sciences ☐ Behavioural & social sciences ☐ Ecological, evolutionary & environmental sciences

For a reference copy of the document with all sections, see nature.com/documents/nr-reporting-summary-flat.pdf

# Life sciences study design

All studies must disclose on these points even when the disclosure is negative.

| | |
|---|---|
| Sample size | No statistical methods were used to pre-determine sample sizes, but our sample sizes are similar to those reported in previous publications [Kim, J., et al. Evolutionarily conserved regulators of tau identify targets for new therapies. Neuron 111, 824-838 e827 (2023).]. The sample size (n) of each experiment is provided in the figure legends. |
| Data exclusions | We didn't excluded any data for analysis unless the experiment technically failed; for example, experiments for which positive control didn't show the expected results. |
| Replication | We performed all experiment in multiple replicates(>= 3) under the same conditions of measurement to verify the reproducibility of our results. For western blots, we used Image Studio program to measure the band intensity, using the same box size for measurements of the same protein band across samples. All set of samples analyzed together, were prepared together and visualized in same gel to exclude variation generated during sample preparation and the western blot assay. For the in vitro seeding assay, we performed it in multiple replicates and we scanned the whole plate automatically using an image scanner. Each set of experiment had multiple number of control samples for normalization. Our attempts at replication were successful. |
| Randomization | Our experimental materials were human cell lines and mice. For mice, viral injections were done in newborn pups, thus by the nature of the design, totally randomized as genotypes not known at that point. All samples generated were used in data collection and analysis. For mice, rarely, we couldn't collect all the sample in some case (for example, a dead mouse). We didn't select partial samples to either discriminatingly measure or collect data. This approach allows for a robust understanding of the pathology within the specific population studied, without the need for inferential statistical adjustments that are typically required in randomized controlled trials. If we found bad samples (for example, bad cell condition and health), we didn't initiate experiments such as infection, transfection, or drug treatment. In cases where cell viability |

was affected after treatment, we used multiple number of samples. Similarly, if there is multiple number of unhealthy cells or positive control which didn't work, we eliminated the whole set of samples.

Blinding

All of our experimental samples and mice were designated by a digital number, blinding the experimenter up until the collection and analysis of data.

# Reporting for specific materials, systems and methods

We require information from authors about some types of materials, experimental systems and methods used in many studies. Here, indicate whether each material, system or method listed is relevant to your study. If you are not sure if a list item applies to your research, read the appropriate section before selecting a response.

## Materials & experimental systems

| n/a | Involved in the study |
|---|---|
| ☐ | ☒ Antibodies |
| ☐ | ☒ Eukaryotic cell lines |
| ☒ | ☐ Palaeontology and archaeology |
| ☐ | ☒ Animals and other organisms |
| ☒ | ☐ Clinical data |
| ☒ | ☐ Dual use research of concern |
| ☒ | ☐ Plants |

## Methods

| n/a | Involved in the study |
|---|---|
| ☒ | ☐ ChIP-seq |
| ☐ | ☒ Flow cytometry |
| ☒ | ☐ MRI-based neuroimaging |

## Antibodies

Antibodies used

Primary antibodies: anti-TYK2 (Abcam, Cat# ab303500, 1:1000), anti- phospho-TYK2(p292) (Abcam, Cat# ab138394, 1:1000), PHF1 (Peter Davies, 1:2000) , anti-tau (Abcam, Cat# ab80579, 1:4000), anti-tau (Agilent, Cat# A0024, 1:10,000), anti-HA (Biolegend, Cat# 901514, 1:4000), anti- Flag (M2) (Sigma, Cat# F3156, 1:4000), anti-p-tau (pT205) (Thermo Fisher Scientific, Cat# 44-738G, 1:2000), anti-tau, oliogomeric (Millipore, Cat# ABN454-I, 1:1000), anti-phospho-Tyr (Millipore, Cat# 05-321, 1:1000), anti-FYN (Cell signaling, Cat# 4023, Technology 1:1000), anit- Vinculin (Sigma, Cat# V9131, 1:2,0000), anti-GAPDH (ImmunoChemical, Cat# 2-RGM2, 1:20,000), anti-GFAP (Norus Biological , Cat# 53809, 1:1000) and anti-IBA1 (Wako, Cat# 019-19741, 1:1,000), anti-phospho-tau (pTyr29) (N/A 1:1000), anti-phospho-tau (pTyr18) ( MediaMab, Cat# MM-0194-P, 1:1000), anti-phopsho-tau (pS396), PHF13 (Cell Signaling Technology, Cat# 9632S, 1:1000), anti-DUSP (ABclonal, Cat# A2919, 1:1000), anti-myc tag antibody (Sigma-Aldrich, Cat# C3956, 1:2000), anti-beta-catenin ( Cell Signaling Technology, Cat#8480, 1:1000)
Secondary antibody :Donkey anti-Rabbit IgG, Alexa Fluor 555 (Thermo Scientific, Cat# A-31572. 1:500), Donkey anti-goat IgG, Alexa Fluor 594 (Jackson ImmunoResearch, Cat# 705-585-003, 1:500), Donkey anti-Rabbit IgG, IRDye® 800CW (Li-COR Bioscience, Cat# 926-32213, 1:10,000), Goat anti-Rabbit IgG, IRDye®680RD(Li-COR Bioscience, Cat# 926-68071, 1:10,000), Goat anti-Mouse IgG, IRDye® 800CW i-COR Bioscience, Cat# 926-32210, 1:10,000), Goat anti-Mouse IgG, IRDye®680RD (Li-COR Bioscience, Cat# 926-68072, 1:10,000)

Validation

1. Mouse anti-tau (tau 5), Abcam, Cat# ab80579: We used this antibody to detect total tau protein in western blots from mouse brain tissues (Figure 1b,1c, and 1d, Figure 2e, 2g and 2h, Figure 4e, Figure 5e, Figure 6a, Figure 7b, Figure 8c, Extended Data Figure 2e, and Extended Data Figure 3a, 3c, Extended Data Figure 4) and immunoprecipitation (IP) samples. This antibody was consistently validated for western blots and immunofluorescence staining of mouse brain tissues in many studies including our previous studies. We confirmed the signal reduction by tau shRNA treatment in western blot analysis. After IP, tau signal was also confirmed by western blot. Applications of this antibody are well described in the manufacturer's website; https://www.abcam.com/products/primary-antibodies/tau-antibody-tau-5-bsa-and-azide-free-ab80579.html

2. Rabbit anti-tau (Dako) Agilent Cat# A0024. We used this antibody to detect total tau protein in human cell lines and mouse brain tissues in western blot assays (Figure 2a, 2b, 2f, Figure 3a-d, Figure 4e, Figure 6a, Figure 7d, Extended Data Figure 1a-c, Extended Data Figure 2a, Extended Data Figure 3d). website; https://www.citeab.com/antibodies/3382933-a0024-tau

3. Mouse anti-HA (HA-7), Biolegend, Cat# 901514: We used this antibody to detect HA-tagged protein including ubiquitinated protein in western blot assays (Figure 2d-f, Figure 3a-c, Extended Data Figure 2c). Website: https://www.biolegend.com/en-us/products/anti-ha-11-epitope-tag-antibody-11071

4. Mouse anti-FLAG (M2), Sigma-Aldrich, Cat# F3165; We used this antibody to detect flag-tagged protein in western blot assays (Figure 2a, 2d, 2e, Figure 3a-c, Figure 4e, Figure 6a, Figure 7b, 7f, Extended Data Figure 2a, 2c, 2d, Extended Data Figure 3c, Extended Data Figure 4), in ICH (Extended Data Figure 3b) and IP experiments to pull down Flag tagged protein in human cell lysates. We confirmed that this antibody detect nothing in western blotting of lysate or IP product from cells without Flag tag expression. Website: https://www.sigmaaldrich.com/US/en/product/sigma/f3165

5. Mouse anti-Phospho-tau (Ser396/Ser404) PHF1 were kindly donated by Dr. Peter Davies. We used this antibody to detected the phosphorylated tau on Ser396/Ser404 residues in western blot assays from mouse brain tissues (Figure 4a, 4d, Figure 7d, 7e, 7f, Figure 8cii) This antibody is well validated for western blot and immunostaining in cells and brain tissues from human or rodent origin. This antibody has been used in a number of publications including our previous papers (Evolutionarily conserved regulators of tau identify targets for new therapies, 2023 [neuron], Reduction of Nuak1 Decreases Tau and Reverses Phenotypes in a Tauopathy Mouse Model, 2016 [neuron]

6. Rabbit anti-tau (T22), oligomeric , EMD Millipore, Cat# ABN454-I; We used this antibody to detect oligomeric tau species in western blot assays of mouse brain tissues (Asai, H., et al. (2015). Nat. Neurosci. 18(11):1584-1593)(Figure 8ciii). This antibody can also be used in dot blot analysis according to manufacturer's website (Blair, L.J., et al. (2013). J. Clin. Invest. 123(10):4158-4169). Website:https://www.sigmaaldrich.com/US/ko/product/mm/abn454i

7. Rabbit anti-Phophso-tau (Thr205), Thermo Fisher Scientific, Cat# 44-738G: This antibody was used to detected phosphorylated tau on Thr205 residue in western blot assays of mouse brain tissues (Figure 8c iv). The manufacturer's website cites 16 published figures including western blots and images of stained cells or stained brain tissues. Applications of this antibody are WB, IHC, IHC(P), IHC(F), ICC/IF, ELISA and DB. The website also cites 38 references. Website: https://www.thermofisher.com/antibody/product/Phospho-Tau-Thr205-Antibody-Polyclonal/44-738G

8. Mouse anti-phosphotyrosine 4G10, EMD Millipore, Cat# 05-321: We used this antibody to detect tyrosine phosphorylated proteins in human cell lysates (Figure 2a, 2d, Figure 7b). This antibody can detect tyrosine phosphoylated proteins in all species. We validated this antibody by finding an increased signal after treating cells with a tyrosine phosphatase inhibitor treatment in a western blot assay. This unique monoclonal antibody is validated for use in IC, IH, IP, WB and backed by hundreds of publications. Website: https://www.merckmillipore.com/KR/ko/product/Anti-Phosphotyrosine-Antibody-clone-4G10,MM_NF-05-321#

9. Rabbit anti-FYN, Cell Signaling Technology, Cat# 4023: We used this antibody to detect FYN kinase in western blot assays from human cells (Extended Data Figure 3d, Extended Data Figure 4). We valiated this antibody by showing a reduced signal in cells with FYN shRNA treatment or increased signal in cell overexpressing FYN kinase protein. Website: https://www.cellsignal.com/products/primary-antibodies/p16-ink4a-bc42-mouse-mab/68410?utm_region=global_hq&utm_tactic=paid&utm_conv=traffic_driver_tdr&utm_source=google&utm_medium=ppc&utm_campaign=can&utm_content=23-can-86063&gclid=CjwKCAjw6eWnBhAKEiwADpnw9rgAN4hgSHMwI0D-Fv-47WHzoiPcyfM1Xlji0f-8K0aer5uTUJIdmhoCdvAQAvD_BwE&gclsrc=aw.ds&_requestid=639377

10. Mouse anti-Vinculin, Sigma-Aldrich Cat# V9131. We used this antibody to detect Vinculin protein in western blot assays of human cell lysates or mouse brain lysates. This antibody is validated for western blotting, immunofluorescence staining and immunohistochemistry. The manufacture website also cites 38 references using this antibody. Website: https://www.sigmaaldrich.com/US/en/product/sigma/v9131

11.Mouse anti-GAPDH, Advanced ImmunoChemical, Cat# 2-RGM2: This antibody has used to detect GAPDH protein in mouse brain lysates or human cell lysates using western blot assays. Based on manufactur's website, this antibody is validated for applications of immunoassay, western blotting, and immunocytochemistry. Website: https://www.advimmuno.com/product/monoclonal-mouse-anti-rabbit-glyceraldehyde-3-phosphate-dehydrogenase-gapdh/

12.Mouse anti-GFAP, Novus Biologicals, Cat# 53809: We used this antibody to detect astrocytes in mouse brain tissue by immunofluorescence staining (Figure 8e). We validated this antibody by comparing staining between the aged WT mouse and the age matched tauopathy mouse model which displays an increased activated astrocytes signal. Validated applications of this antibody are WB, IHC, IHC(P), IHC(F), and ELISA, as per the manufacturer's website. Website: https://www.novusbio.com/products/gfap-antibody_nb100-53809

13. Mouse anti-Iba1, Wako Chemicals, Cat# 019-19741: We used this antibody to detect microglia in mouse brain tissue by immunofluorescence staining (Figure 8e). We validated this antibody by comparing staining between the aged WT mouse and the age matched tauopathy mouse model which displays an increased activated microglia signal. This antibody was validated for ICC and IHC(Frozen). Manufacture website cites 3,879 publications which used this antibody.

14. Rabbit Phophso-tau (pY29): This antibody used to detect phosphorylated Tyrosine 29 residue of tau (Figure 4e, Figure 6a, Figure 7b, Extended Data Figure 3a,3c, Extended Data Figure 4). This antibody was generated in our laboratory and validated by western blot and Immunocytochemistry (Extended data Figure 3) showing specifically Tyrosine29 phosphoryllated tau in the presence of TYK2 but not with our Mock (RFP, transfection control) expression.

15. Mouse anti-TYK2: This antibody used to detect human TYK2 protein in human brain samples in western blot analysis (Figure 4a). Based on manufacture's website, this antibody is suitable for WB, IP and reacts with Human, Mouse, Rat samples. Website: https://www.abcam.com/en-us/products/primary-antibodies/tyk2-antibody-epr24628-106-ab303500

16. Rabbit anti-phospho-TYK2 (p292): We used this antibody to detect activated TYK2 in human brain samples in western blot analysis (Figure 4d_ Based on manufacture's website, this antibody is suitable for WB and reacts with Human samples. The manufacture website also cites 1 references using this antibody. Website: https://www.abcam.com/en-us/products/primary-antibodies/tyk2-phospho-y292-antibody-ab138394

17. Mouse anti-Phospho-tau (pY18): We used this antibody to detect phosphorylated tau at Tyr18 residue in western blot analysis (Figure 2f and Extended Data Figure 3d ). We validated this antibody to work properly in western blotting analysis by showing higher antibody staining in cell samples in the presence of FYN kinase (phosphorylates tau at Tyr18 residue) comparing to RFP overexpressing control samples. Based on manufacture's website, this antibody is suitable for IF, IHC, and WB and react with Human, Mouse and Rat samples. Website: https://medimabs.com/product/phospho-tau-tyr-18-mouse-monoclonal-antibody-9g3/

18. Mouse anti-Phospho-tau (pS396), PHF13: We used this antibody to detect phosphorylated tau at Ser396 in western blot anaylsis (Extended Data Figure 4). We validated this antibody by comparing phosphorylated tau signal between samples which express GSK3beta (phosphorylates tau at multiple residues including Ser396) or RFP control. Base on manufacture's website, this antibody is validated for WB, and reacts with Human, Mouse, Rat samples. Manufacture website cites 74 publications which used this antibody. Website: https://www.cellsignal.com/products/primary-antibodies/phospho-tau-ser396-phf13-mouse-mab/9632

19. Rabbit anti-DUSP1: This antibody was used to detect human DUSP1 protein in western blot to validated the knocking down of DUSP1 by DUSP1-shRNA treatment in human cell culture (Extended Data Figure 3c and 3d) . Based on manufacture's website, this antibody is validated for WB and ELISA and reacts with Human, Mouse, Rat samples. Website: https://abclonal.com/catalog-antibodies/DUSP1MKP1RabbitpAb/A2919#section8

20. Mouse anti-myc tag: This antibody was used to detect the myc-tagged protein in western blot analysis (Figure a-c, Extended Data Figure 4), Based on manufacture's website, this antibody is validated for WB, IP, ICH, and suitable for array experiment.
Website :https://www.sigmaaldrich.com/US/en/product/sigma/c3956

21. Rabbit anti-beta-catenin: This antibody was used to detect endogenous beta-catenin in human cell line (Figure 3d). Based on manufacture's website, this antibody is suitable for IP WB, ICH, IF, F, ChIP, C&R, and reacts with Human, Mouse, Rat, Monkey samples. Website: https://www.cellsignal.com/products/primary-antibodies/b-catenin-d10a8-xp-rabbit-mab/8480

## Eukaryotic cell lines

Policy information about cell lines and Sex and Gender in Research

| | |
|---|---|
| Cell line source(s) | HEK293T ATCC CRL-3216; RRID:CVCL_0063<br>Tau RD P301S PRET Biosenser ATCC CRL-3275<br>Neuro-2a ATCC CCL-131<br>SHSY5Y ATCC CRL-2266 |
| Authentication | None of these cell lines have been authenticated. |
| Mycoplasma contamination | HEK293T for transfection or infection: not tested for mycoplasma contamination.<br>Neuro-2a, SHSY5Y for tranfection and infection : not tested mycoplasma contamination.<br>Tau RD P301S PRET Biosenser: not tested mycoplasma contamination.<br>HEK293T for AAV production: tested negative for mycoplasma contamination |
| Commonly misidentified lines<br>(See ICLAC register) | There is no commonly misidentified lines in this study. |

## Animals and other research organisms

Policy information about studies involving animals; ARRIVE guidelines recommended for reporting animal research, and Sex and Gender in Research

| | |
|---|---|
| Laboratory animals | CFW (8 week~ 6months old), FBV (8 week~ 6months old), B6;C3-Tg(Prnp-MAPT*P301S)PS19Vle/J(1~9 months old), B10.D1-H2q/SgJ (Allele Symbol Tyk2E775K, 1 month old), C57BL/6N-Tyk2tm2b(EUCOMM)Hmgu/Ieg (1 month old) |
| Wild animals | No wild animals were used in this study. |
| Reporting on sex | In this study, we used both male and female of rodents.We used mice of both sex for data collection and we tried to collect data from equal number of each sex. For western blot data, we collected tissues from 15-30 animals. For immunoflourscence experiments, we stained tissues from 6-8 animals. For histopathology we did not find any sex differences. For western blot data, we normalized each data by sex then analyzed. We did this because male and female are known to show different degrees of pathology. We confirmed that there is no sex-differences after normalization in our results. |
| Field-collected samples | No field-collected animals were used in this study. |
| Ethics oversight | All procedures for mice work were reviewed and approved by the Baylor College of Medicine Institutional Animal Care and Use Committee(IACUC) in accordance with the guidelines of the US National Institutes of Health. |

Note that full information on the approval of the study protocol must also be provided in the manuscript.

## Plants

| | |
|---|---|
| Seed stocks | None |
| Novel plant genotypes | None |
| Authentication | *Describe any authentication procedures for each seed stock used or novel genotype generated. Describe any experiments used to assess the effect of a mutation and, where applicable, how potential secondary effects (e.g. second site T-DNA insertions, mosiacism, off-target gene editing) were examined.* |

## Flow Cytometry

### Plots

Confirm that:

☒ The axis labels state the marker and fluorochrome used (e.g. CD4-FITC).

☒ The axis scales are clearly visible. Include numbers along axes only for bottom left plot of group (a 'group' is an analysis of identical markers).

☒ All plots are contour plots with outliers or pseudocolor plots.

☒ A numerical value for number of cells or percentage (with statistics) is provided.

### Methodology

| | |
|---|---|
| Sample preparation | Tau-seeded transduction of tau RD P301S FRET Biosensor cell line and flow cytometry analysis were conducted as previously described with minor modifications. The biosensor cells were plated in 96-well plates at a density of 20,000 cells/well. After 24h, when cells reached 60-70% confluency, we transduced tau seeds complexes by combining [15 micro-litter Opti-MEM (Gibco, 31985070) + 0.1-0..4 micro-litter Lipofectamine 2000 (Invitrogen, 11668-500)] with [7.5 micro-litter Opti-MEM + 0.5-2.0 micro-gram protein extract from brain homogenate from PS19 mice]. The transduction complexes were then incubated for 30 min at room temperature (RT) and added to cells. After 48-72 h of seed transduction, cells were dissociated with trypsin (Life Technologies, # 25200072), and resuspended in 1x PBS containing 2 mM EDTA and 3% FBS. |
| Instrument | BD LSRFortessa™ cell analyzer Cat. No. 649225 |
| Software | Collection: BD FACS Diva (v9.0) |
| Cell population abundance | We collected around 100,000-120,000 events and got alive singlets using FSC-A/SSC (x/y axis) and FSC-A/FSC-H from total events . Then we measured the FRET signal by recording CFP and FRET-YFP signals of 80000-100,000 singlet events at 485/22nm-filter and 525/50 nm-filter, respectively. Seeding activity of given brain homogenates was assessed by the percentage of FRET-positive cells in a bivariate plot of FRET vs. CFP. Typical cell populations at each gating stage are as follows:<br>Cells: 70%-90%, Single cells: 70%-90%, FRET: 0-10% |
| Gating strategy | We collected around 100,000-120,000 events per sample. We used FSC-A (0-250[x1,000])/SSC (0-250 [x1.000])(x/y axis) to select alive cells. Then for single cells, we used FSC-A (0-250[x1,000])/FSC-H (0-250 [x1.000]). For FRET signal, we used Alexa Fluor 405-A[0-10^5]/Horizon V500-A[0-10^5]. Seeding activity of given brain homogenates was assessed by the percentage of FRET-positive cell in a bivariate plot of FRET vs. CFP. In this bivariate plot, to assess the number of FRET-positive cells, we created a gate by using the FRET-negative signal exhibited by biosensor cells treated with lipofectamine alone and the FRET-positive signal exhibited by HEK293T cells expressing CFP fused with YFP. |

☒ Tick this box to confirm that a figure exemplifying the gating strategy is provided in the Supplementary Information.
