# Supplementary figures and images for: TYK2 regulates tau levels, phosphorylation and aggregation in a tauopathy mouse model
Source: Nat Neurosci. 2024 Nov 11;27(12):2417–29. doi: 10.1038/s41593-024-01777-2 (PMC11614740; doi:10.1038/s41593-024-01777-2)

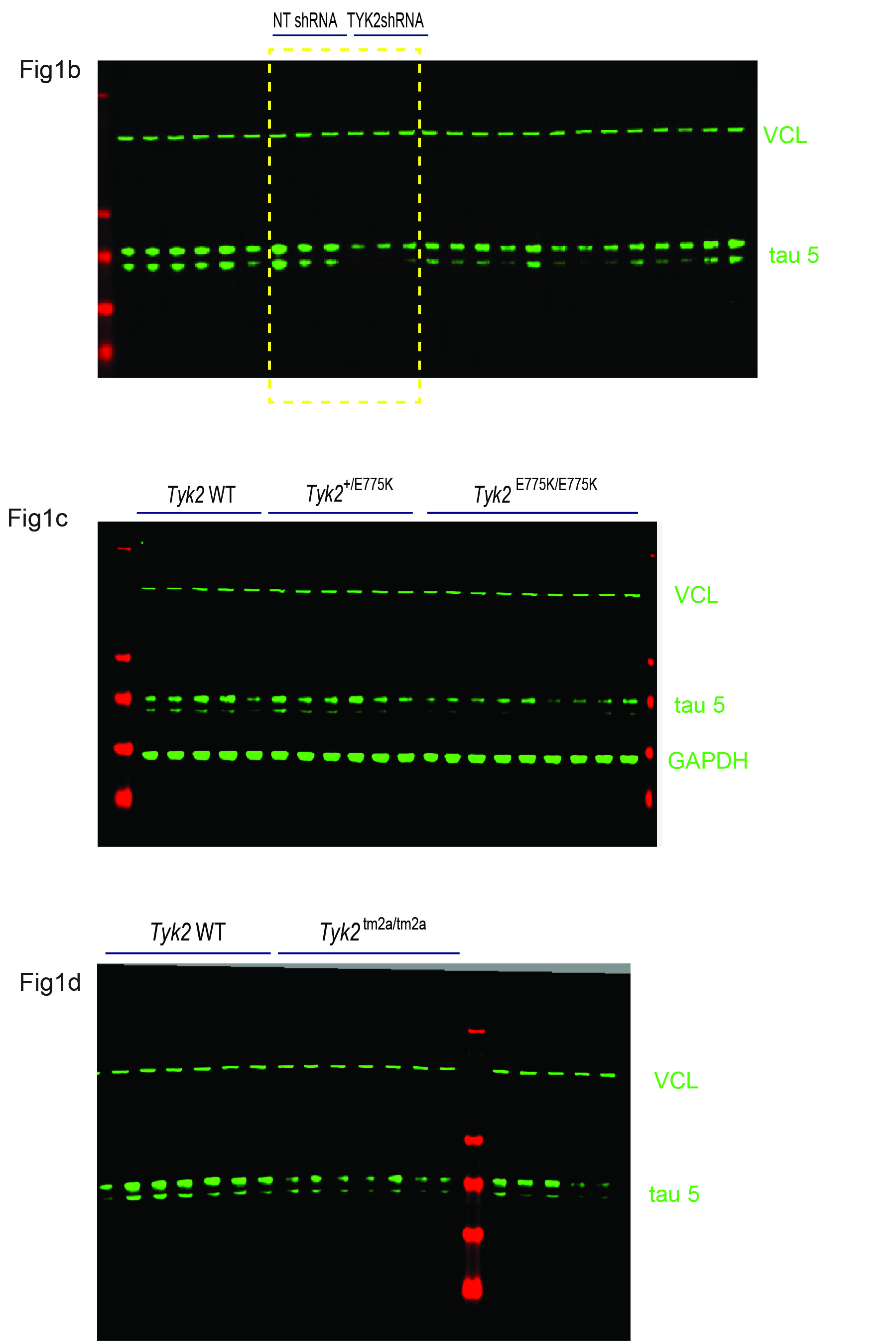

Supplement: Supplementary file 6 — Unprocessed western blots. [file 41593_2024_1777_MOESM6_ESM.jpg]

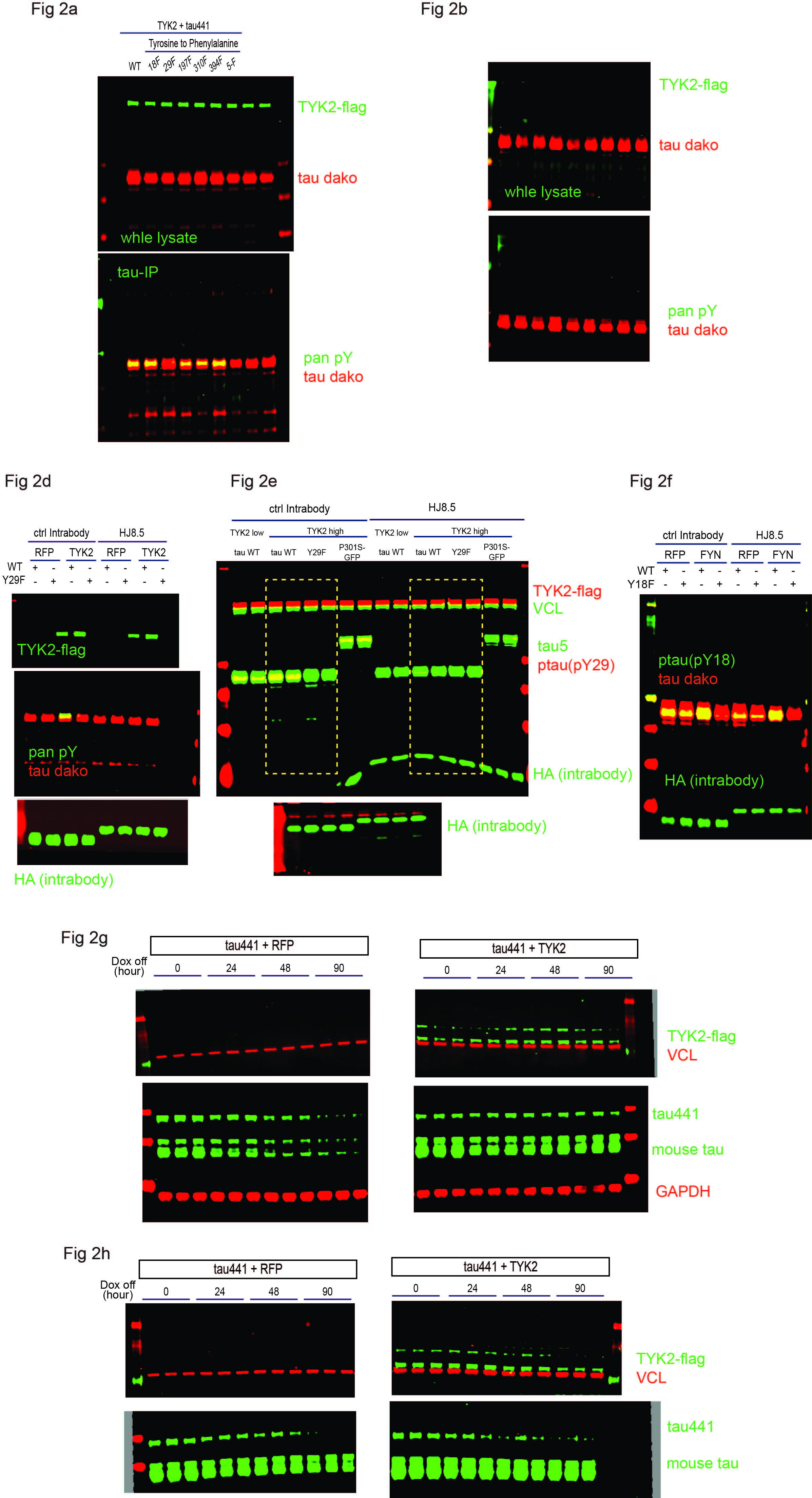

Supplement: Supplementary file 7 — Unprocessed western blots. [file 41593_2024_1777_MOESM7_ESM.jpg]

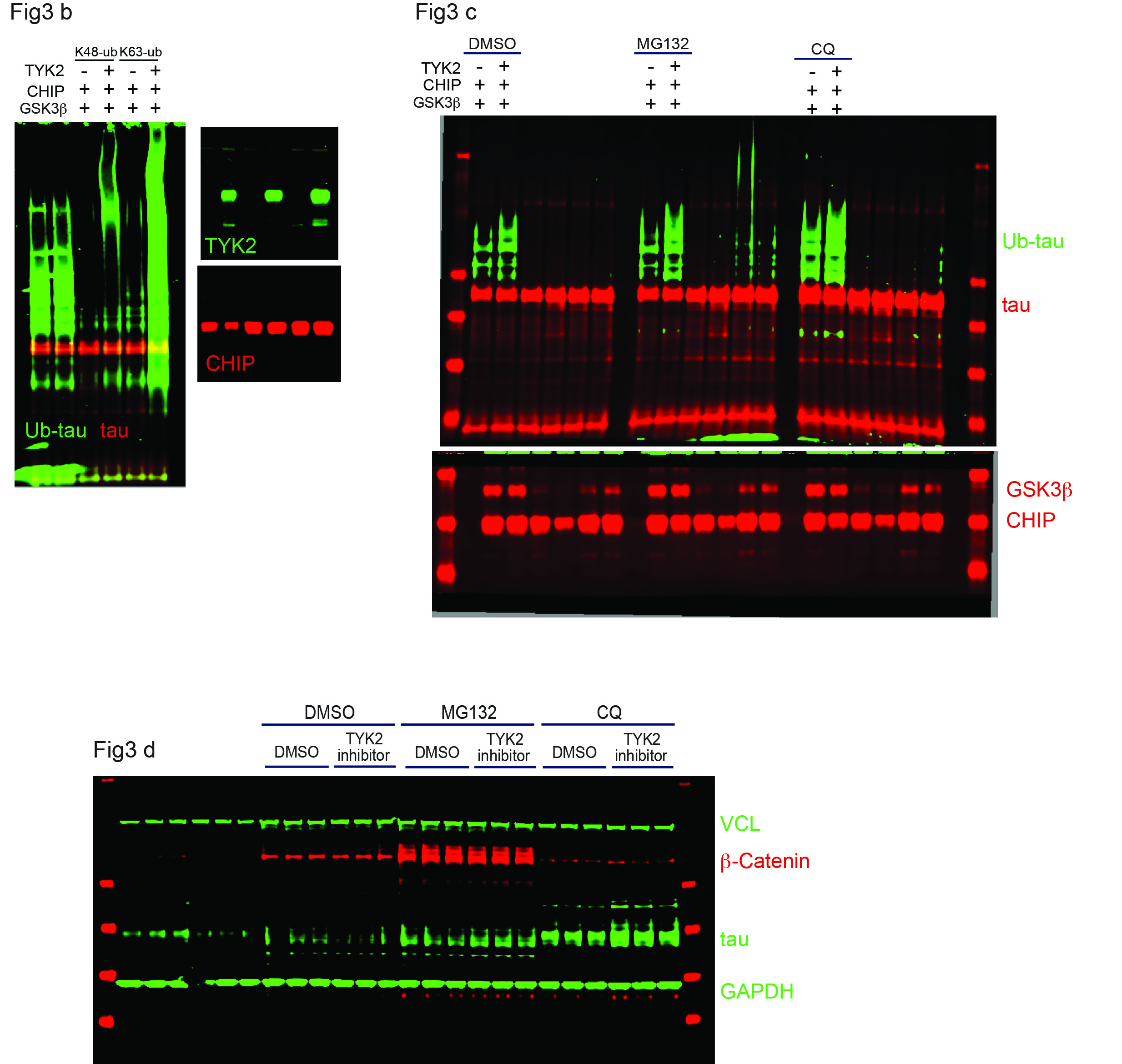

Supplement: Supplementary file 8 — Unprocessed western blots. [file 41593_2024_1777_MOESM8_ESM.jpg]

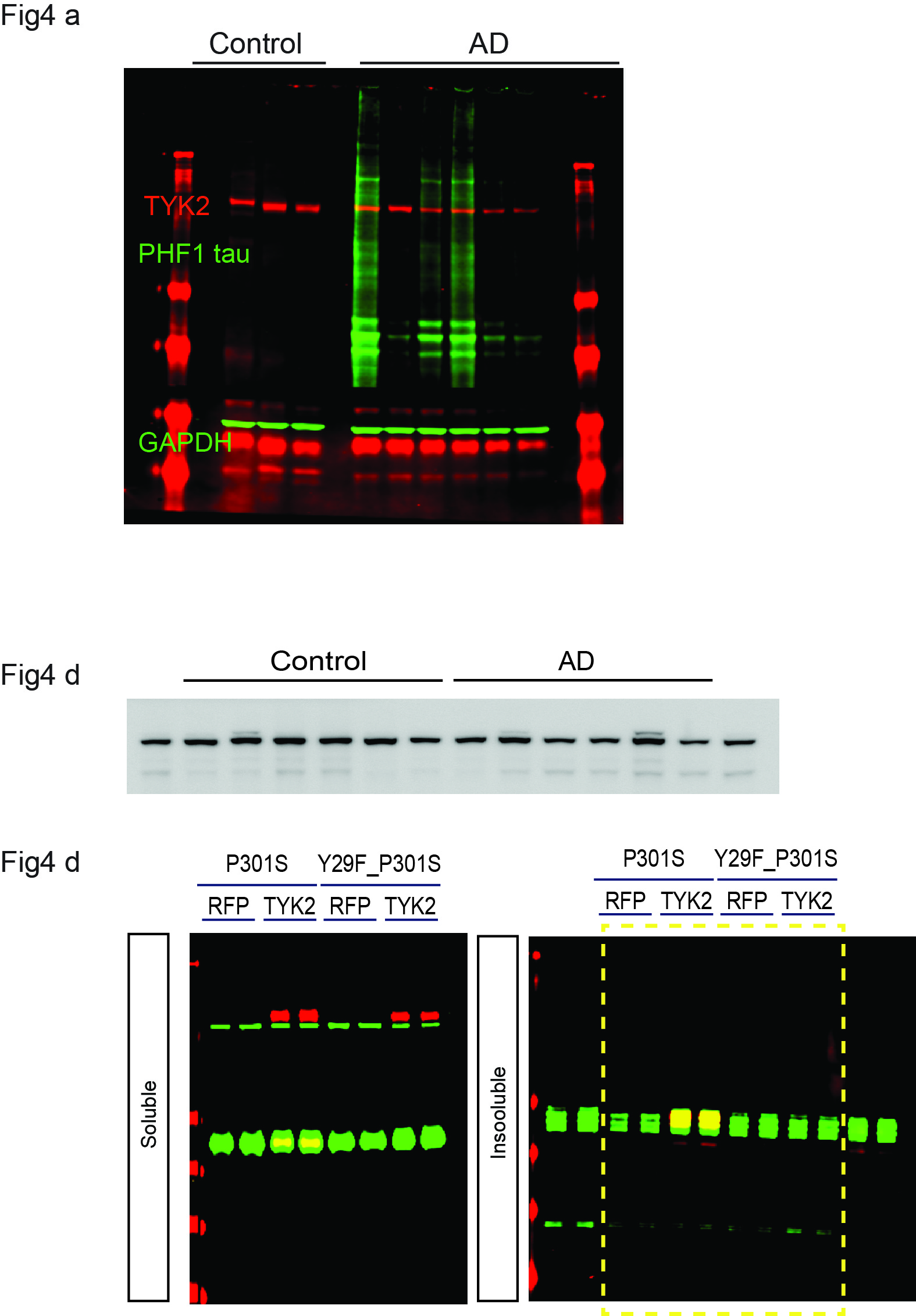

Supplement: Supplementary file 9 — Unprocessed western blots. [file 41593_2024_1777_MOESM9_ESM.jpg]

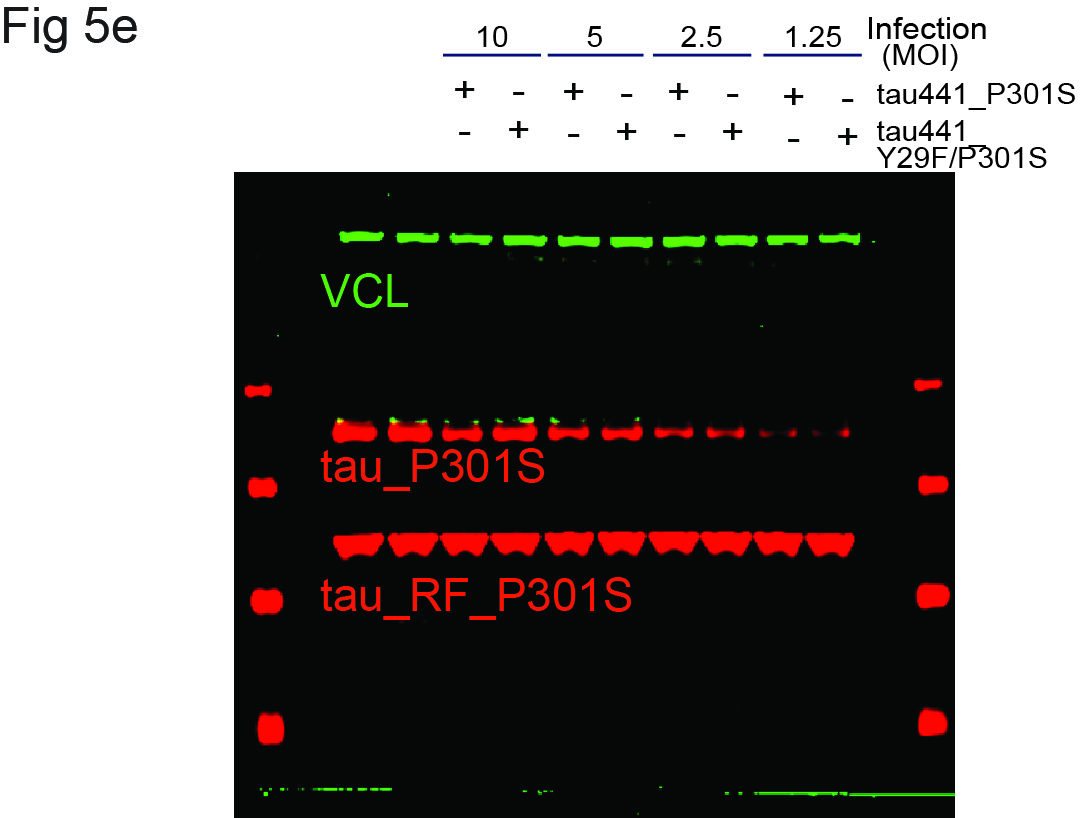

Supplement: Supplementary file 10 — Unprocessed western blots. [file 41593_2024_1777_MOESM10_ESM.jpg]

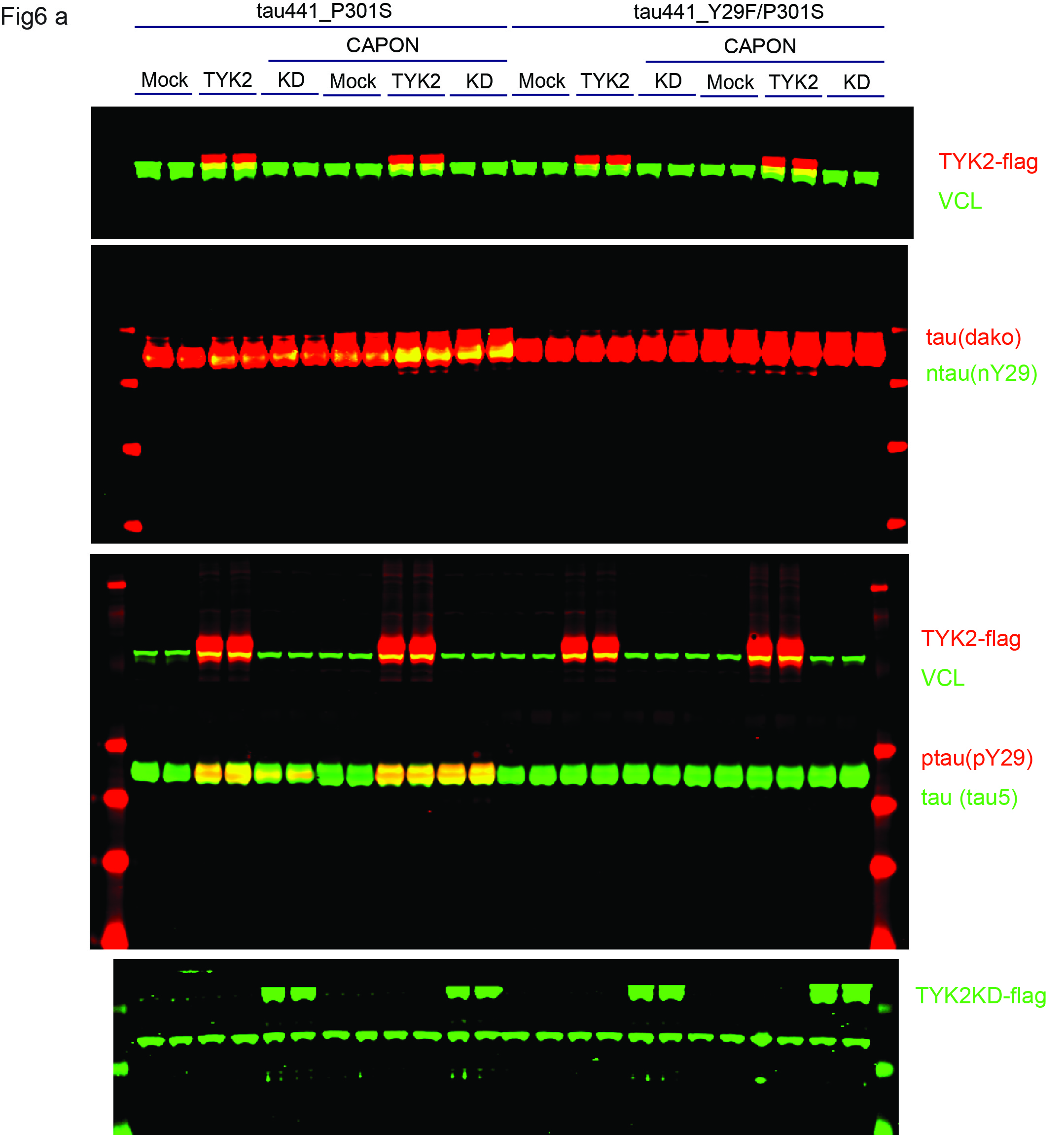

Supplement: Supplementary file 11 — Unprocessed western blots. [file 41593_2024_1777_MOESM11_ESM.jpg]

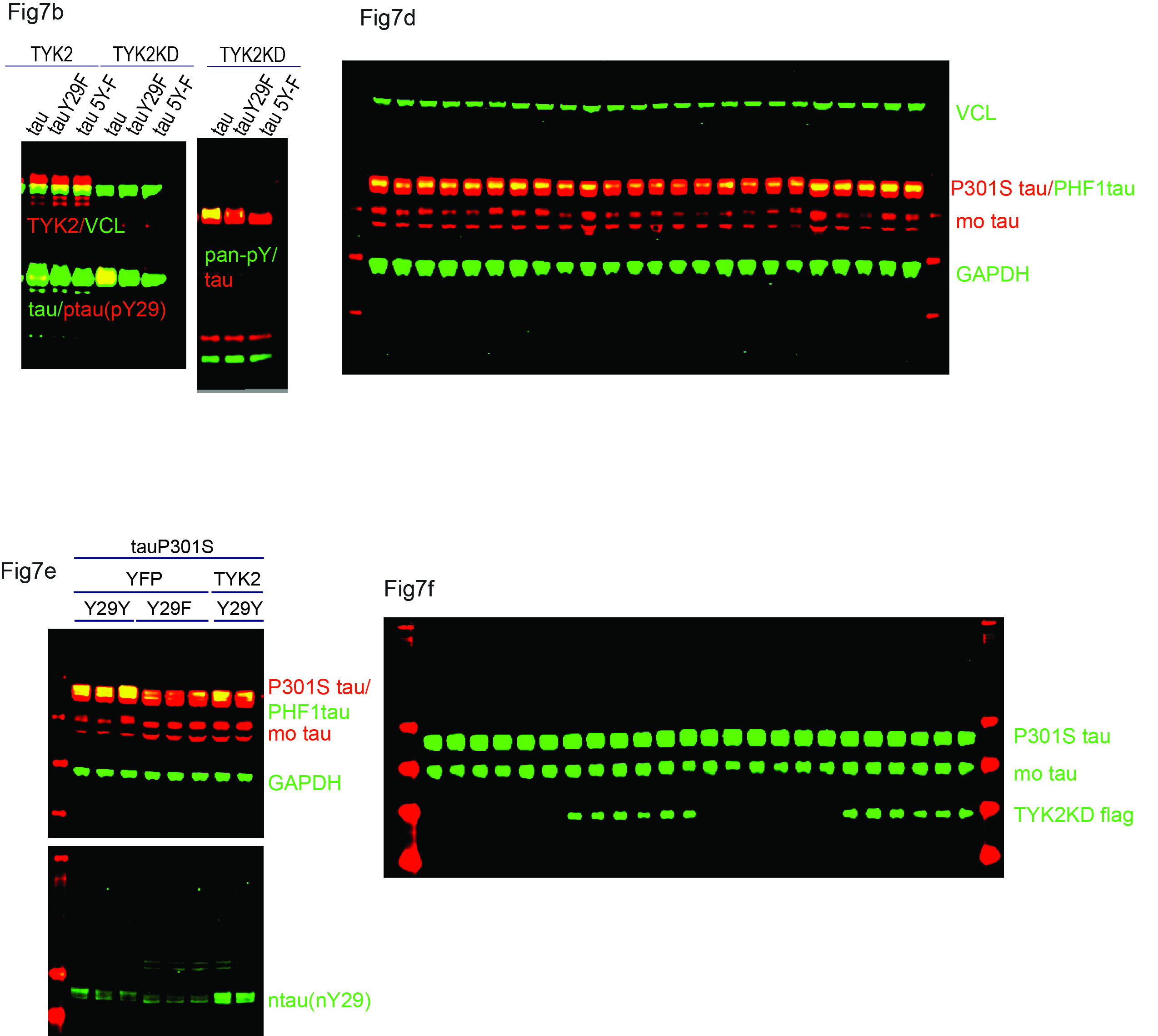

Supplement: Supplementary file 12 — Unprocessed western blots. [file 41593_2024_1777_MOESM12_ESM.jpg]

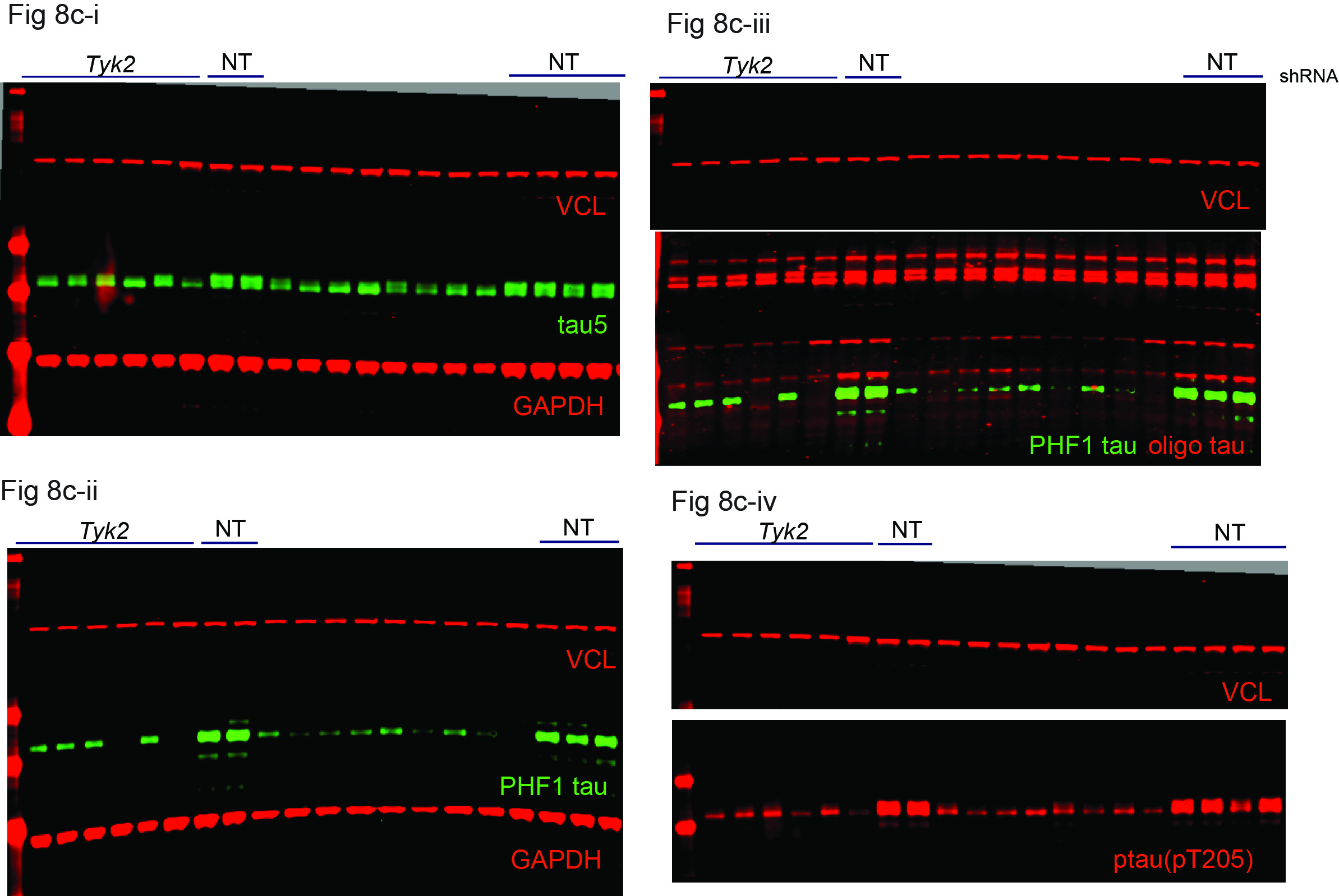

Supplement: Supplementary file 13 — Unprocessed western blots. [file 41593_2024_1777_MOESM13_ESM.jpg]

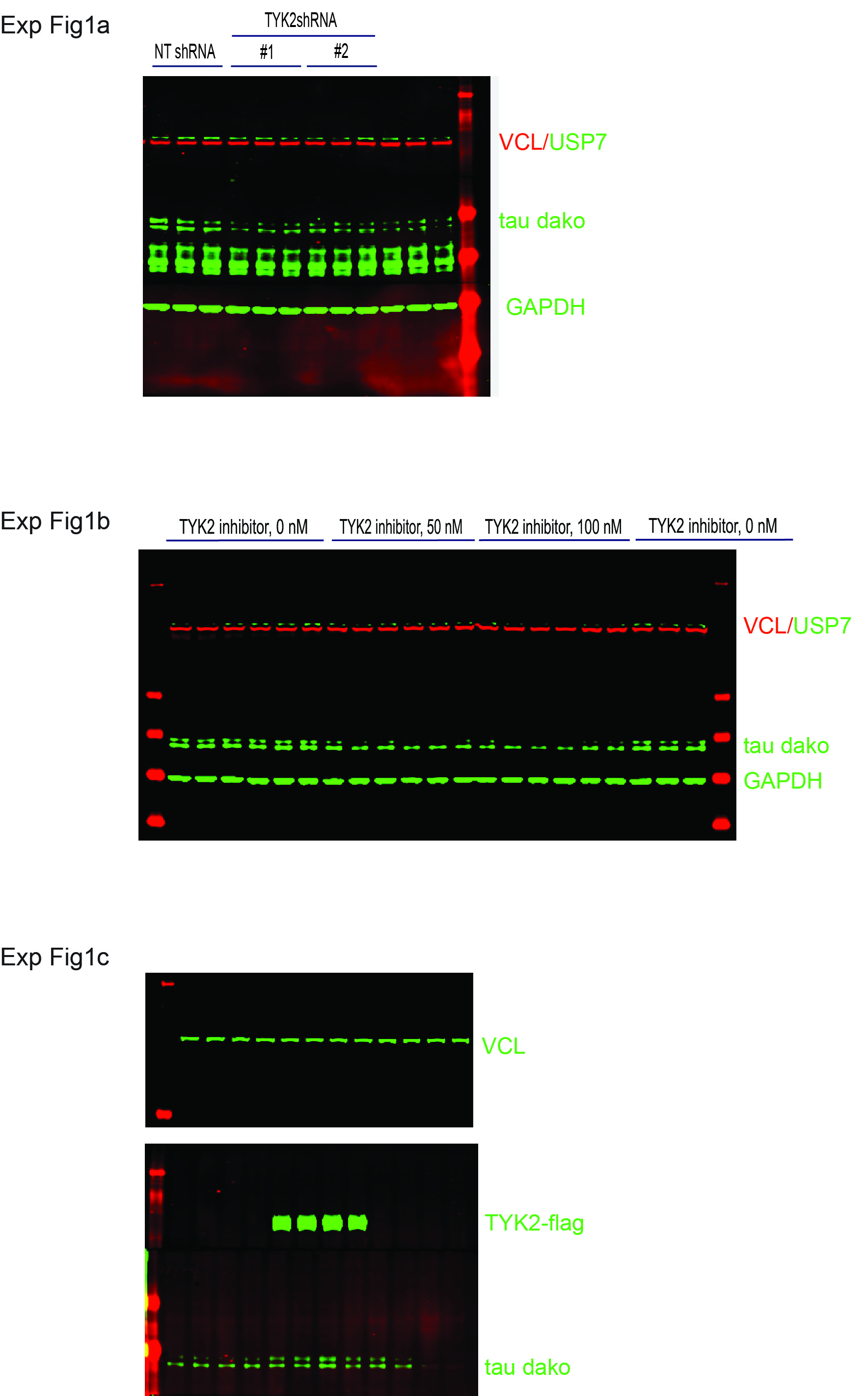

Supplement: Supplementary file 14 — Unprocessed western blots. [file 41593_2024_1777_MOESM14_ESM.jpg]

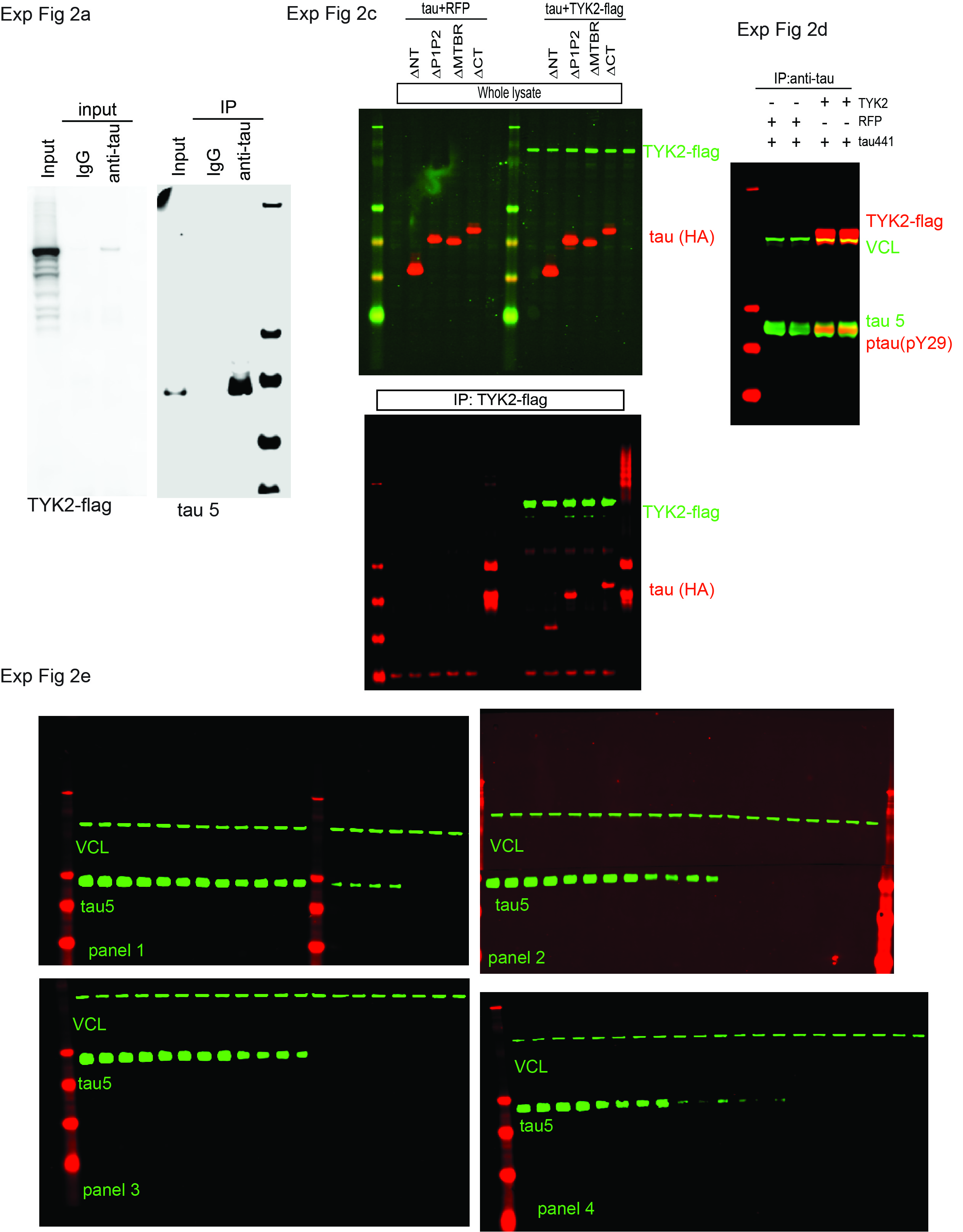

Supplement: Supplementary file 15 — Unprocessed western blots. [file 41593_2024_1777_MOESM15_ESM.jpg]

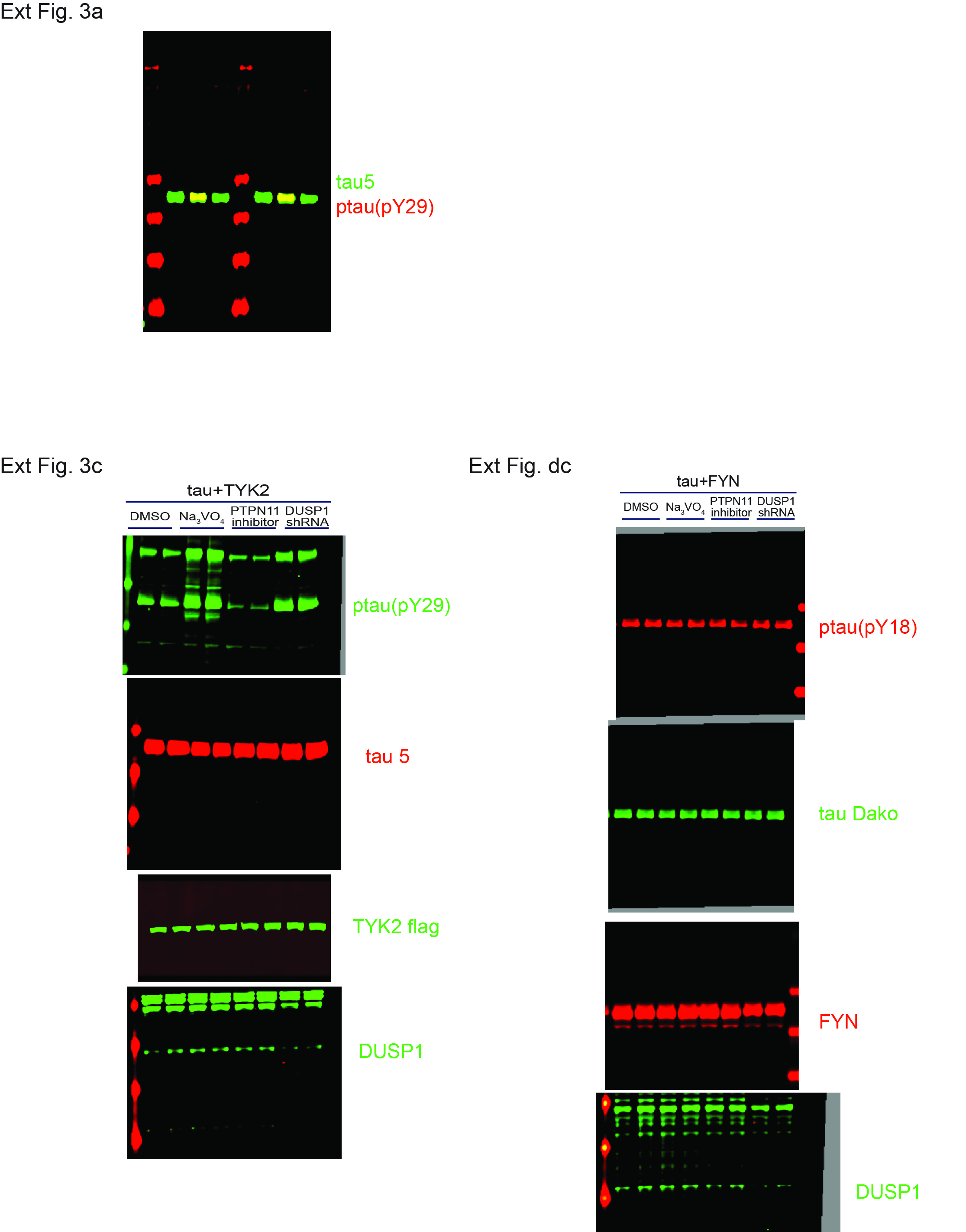

Supplement: Supplementary file 16 — Unprocessed western blots. [file 41593_2024_1777_MOESM16_ESM.jpg]

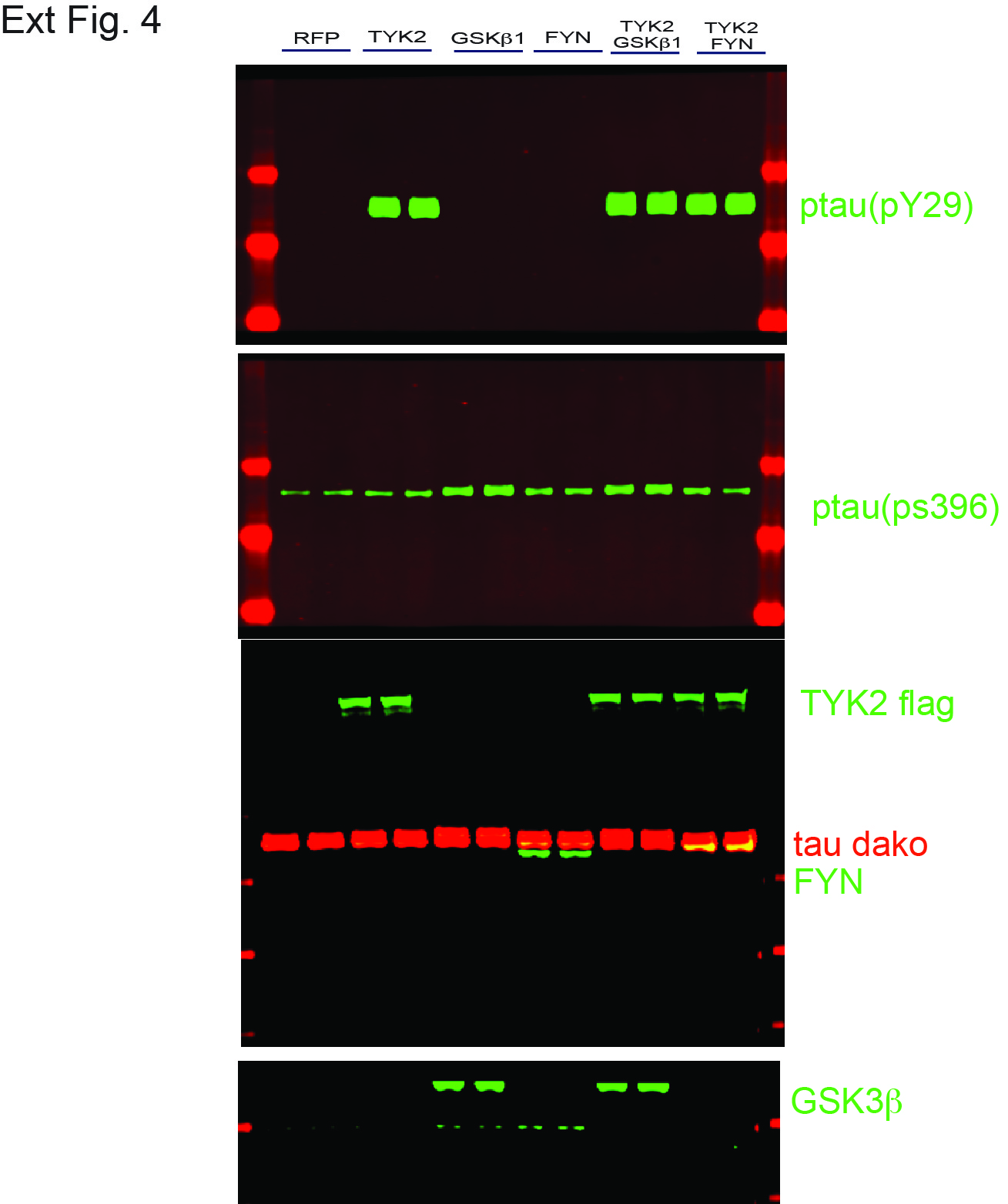

Supplement: Supplementary file 17 — Unprocessed western blots. [file 41593_2024_1777_MOESM17_ESM.jpg]
